# Data Assimilation Methods for Neuronal State and Parameter Estimation

**DOI:** 10.1186/s13408-018-0066-8

**Published:** 2018-08-09

**Authors:** Matthew J. Moye, Casey O. Diekman

**Affiliations:** Department of Mathematical Sciences & Institute for Brain and Neuroscience Research, New Jersey Institute of Technology, Newark, USA

**Keywords:** Data assimilation, Neuronal excitability, Conductance-based models, Parameter estimation

## Abstract

This tutorial illustrates the use of data assimilation algorithms to estimate unobserved variables and unknown parameters of conductance-based neuronal models. Modern data assimilation (DA) techniques are widely used in climate science and weather prediction, but have only recently begun to be applied in neuroscience. The two main classes of DA techniques are sequential methods and variational methods. We provide computer code implementing basic versions of a method from each class, the Unscented Kalman Filter and 4D-Var, and demonstrate how to use these algorithms to infer several parameters of the Morris–Lecar model from a single voltage trace. Depending on parameters, the Morris–Lecar model exhibits qualitatively different types of neuronal excitability due to changes in the underlying bifurcation structure. We show that when presented with voltage traces from each of the various excitability regimes, the DA methods can identify parameter sets that produce the correct bifurcation structure even with initial parameter guesses that correspond to a different excitability regime. This demonstrates the ability of DA techniques to perform nonlinear state and parameter estimation and introduces the geometric structure of inferred models as a novel qualitative measure of estimation success. We conclude by discussing extensions of these DA algorithms that have appeared in the neuroscience literature.

**Electronic Supplementary Material:**

The online version of this article (10.1186/s13408-018-0066-8) contains supplementary material.

## Introduction

### The Parameter Estimation Problem

The goal of conductance-based modeling is to be able to reproduce, explain, and predict the electrical behavior of a neuron or networks of neurons. Conductance-based modeling of neuronal excitability began in the 1950s with the Hodgkin–Huxley model of action potential generation in the squid giant axon [1]. This modeling framework uses an equivalent circuit representation for the movement of ions across the cell membrane:

1 $$ C\frac{dV}{dt} = I_{\textrm{app}}-\sum_{\textrm{ion}} I_{\textrm{ion}}, $$

where *V* is membrane voltage, *C* is cell capacitance, $I_{ \textrm{ion}}$ are ionic currents, and $I_{\textrm{app}}$ is an external current applied by the experimentalist. The ionic currents arise from channels in the membrane that are voltage- or calcium-gated and selective for particular ions, such sodium (Na^+^) and potassium (K^+^). For example, consider the classical Hodgkin–Huxley currents:

2 $$\begin{aligned} I_{\textrm{Na}} &= g_{\textrm{Na}} m^{3}h (V-E_{\textrm{Na}}), \end{aligned}$$

3 $$\begin{aligned} I_{\textrm{K}} &= g_{\textrm{K}} n^{4}(V-E_{\textrm{K}}) . \end{aligned}$$

The maximal conductance $g_{\textrm{ion}}$ is a parameter that represents the density of channels in the membrane. The term $(V-E_{\textrm{ion}})$ is the driving force, where the equilibrium potential $E_{\textrm{ion}}$ is the voltage at which the concentration of the ion inside and outside of the cell is at steady state. The gating variable *m* is the probability that one of three identical subunits of the sodium channel is “open”, and the gating variable *h* is the probability that a fourth subunit is “inactivated”. Similarly, the gating variable *n* is the probability that one of four identical subunits of the potassium channel is open. For current to flow through the channel, all subunits must be open and not inactivated. The rate at which subunits open, close, inactivate, and de-inactivate depends on the voltage. The dynamics of the gating variables are given by

4 $$ \frac{dx}{dt}= \alpha_{x}(V) (1-x) +\beta_{x}(V)x, $$

where $\alpha_{x}(V)$ and $\beta_{x}(V)$ are nonlinear functions of voltage with several parameters.

The parameters of conductance-based models are typically fit to voltage-clamp recordings. In these experiments, individual ionic currents are isolated using pharmacological blockers and one measures current traces in response to voltage pulses. However, many electrophysiological datasets consist of current-clamp rather than voltage-clamp recordings. In current-clamp, one records a voltage trace (e.g., a series of action potentials) in response to injected current. Fitting a conductance-based model to current-clamp data is challenging because the individual ionic currents have not been measured directly. In terms of the Hodgkin–Huxley model, only one state variable (*V*) has been observed, and the other three state variables (*m*, *h*, and *n*) are unobserved. Conductance-based models of neurons often contain several ionic currents and, therefore, more unobserved gating variables and more unknown or poorly known parameters. For example, a model of HVC neurons in the zebra finch has 9 ionic currents, 12 state variables, and 72 parameters [2]. An additional difficulty in attempting to fit a model to a voltage trace is that if one performs a least-squares minimization between the data and model output, then small differences in the timing of action potentials in the data and the model can result in large error [3]. Data assimilation methods have the potential to overcome these challenges by performing state estimation (of both observed and unobserved states) and parameter estimation simultaneously.

### Data Assimilation

Data assimilation can broadly be considered to be the optimal integration of observations from a system to improve estimates of a model output describing that system. Data assimilation (DA) is used across the geosciences, e.g., in studying land hydrology and ocean currents, as well as studies of climates of other planets [4–6]. An application of DA familiar to the general public is its use in numerical weather prediction [7]. In the earth sciences, the models are typically high-dimensional partial differential equations (PDEs) that incorporate dynamics of the many relevant governing processes, and the state system is a discretization of those PDEs across the spatial domain. These models are nonlinear and chaotic, with interactions of system components across temporal and spatial scales. The observations are sparse in time, contaminated by noise, and only partial with respect to the full state-space.

In neuroscience, models can also be highly nonlinear and potentially chaotic. When dealing with network dynamics or wave propagation, the state-space can be quite large, and there are certainly components of the system for which one would not have time course measurements [8]. As mentioned above, if one has a biophysical model of a single neuron and measurements from a current-clamp protocol, the only quantity in the model that is actually measured is the membrane voltage. The question then becomes: how does one obtain estimates of the full system state?

To begin, we assume we have a model to represent the system of interest and a way to relate observations we have of that system to the components of the model. Additionally, we allow, and naturally expect, there to be errors present in the model and measurements. To start, let us consider first a general model with linear dynamics and a set of discrete observations which depend linearly on the system components:

5 $$\begin{aligned} x_{k+1} &=Fx_{k}+\omega_{k+1} , \quad x_{k} \in\mathbb{R}^{L} \end{aligned}$$

6 $$\begin{aligned} y_{k+1} &=Hx_{k+1}+\eta_{k+1}, \quad y_{k+1} \in\mathbb{R}^{M} . \end{aligned}$$

In this state-space representation, $x_{k}$ is interpreted as the state of the system at some time $t_{k}$, and $y_{k}$ are our observations. For application in neuroscience, we can take $M \ll L$ as few state variables of the system are readily observed. *F* is our model which maps states $x_{k}$ between time points $t_{k}$ and $t_{k+1}$. *H* is our observation operator which describes how we connect our observations $y_{k+1}$ to our state-space at $t_{k+1}$. The random variables $\omega_{k+1}$ and $\eta_{k+1}$ represent model error and measurement error, respectively. A simplifying assumption is that our measurements are diluted by Gaussian white noise, and that the error in the model can be approximated by Gaussian white noise as well. Then $\omega_{k} \sim\mathcal{N}(0,Q_{k})$ and $\eta_{k}\sim\mathcal{N}(0,R_{k})$, where $Q_{k}$ is our model error covariance matrix and $R_{k}$ is our measurement error covariance matrix. We will assume these distributions for the error terms for the remainder of the paper.

We now have defined a stochastic dynamical system where we have characterized the evolution of our states and observations therein based upon assumed error statistics. The goal is now to utilize these transitions to construct methods to best estimate the state *x* over time. To approach this goal, it may be simpler to consider the evaluation of *background* knowledge of the system compared to what we actually observe from a measuring device. Consider the following cost function [9]:

7 $$ C(x) = \frac{1}{2} \lVert y-Hx \rVert^{2}_{R}+ \frac{1}{2} \bigl\lVert x-x^{b} \bigr\rVert ^{2}_{P ^{b}}, $$

where $\lVert z \rVert^{2}_{A} =z^{T}A^{-1}z$. $P^{b}$ acts to give weight to certain background components $x^{b}$, and *R* acts in the same manner to the measurement terms. The model or background term acts to regularize the cost function. Specifically, trying to minimize $\frac{1}{2} \lVert y-Hx \rVert^{2}_{R}$ is underdetermined with respect to the observations unless we can observe the full system, and the model term aims to inform the problem of the unobserved components. We are minimizing over state components *x*. In this way, we balance the influence of what we think we know about the system, such as from a model, compared to what we can actually observe. The cost function is minimized from

8 $$ \nabla C = \bigl(H^{T} R^{-1}H + \bigl(P^{b} \bigr)^{-1} \bigr)x^{a} - \bigl(H^{T}R^{-1}y+ \bigl(P^{b} \bigr)^{-1}x ^{b} \bigr)=0. $$

This can be restructured as

9 $$ x^{a}=x^{b} + K \bigl(y-Hx^{b} \bigr), $$

where

10 $$ K=P^{b}H^{T} \bigl(HP^{b}H^{T}+R \bigr)^{-1}. $$

The optimal Kalman gain matrix *K* acts as a weighting of the confidence of our observations to the confidence of our background information given by the model. If the background uncertainty is relatively high or the measurement uncertainty is relatively low, *K* is larger, which more heavily weights the *innovation* $y-Hx^{b}$.

The solution of (7) can be interpreted as the solution of a single time step in our state-space problem (5)–(6). In the DA literature, minimizing this cost function independent of time is referred to as 3D-Var. However, practically we are interested in problems resembling the following:

11 $$ C(x) = \frac{1}{2}\sum_{k=0}^{N} \lVert y_{k}-Hx_{k} \rVert^{2}_{R_{k}}+ \frac{1}{2}\sum_{k=0}^{N-1} \lVert x_{k+1}-Fx_{k} \rVert^{2}_{P^{b}_{k}}, $$

where formally the background component $x^{b}$ has now been replaced with our model. Now we are concerned with minimizing over an observation window with $N+1$ time points. *Variational methods*, specifically “weak 4D-Var”, seek minima of (11) either by formulation of an adjoint problem [10], or directly from numerical optimization techniques.

Alternatively, *sequential data assimilation* approaches, specifically *filters*, aim to use information from previous time points $t_{0},t_{1},\ldots ,t_{k}$, and observations at the current time $t_{k+1}$, to optimally estimate the state at $t_{k+1}$. The classical Kalman filter utilizes the form of (10), which minimizes the trace of the posterior covariance matrix of the system at step $k+1$, $P_{k+1}^{a}$, to update the state estimate and system uncertainty.

The Kalman filtering algorithm takes the following form. Our *analysis* estimate, $\hat{x}_{k}^{a}$ from the previous iteration, is mapped through the linear model operator *F* to obtain our *forecast* estimate $\hat{x}^{f}_{k+1}$:

12 $$ \hat{x}^{f}_{k+1}=F_{k} \hat{x}_{k}^{a}. $$

The observation operator *H* is applied to the forecast estimate to generate the measurement estimate $\hat{y}^{f}_{k+1}$:

13 $$ \hat{y}^{f}_{k+1}=H_{k+1}\hat{x}^{f}_{k+1}. $$

The forecast estimate covariance $P^{f}_{k+1}$ is generated through calculating the covariance from the model and adding it with the model error covariance $Q_{k}$:

14 $$ P^{f}_{k+1} = F_{k}P_{k}^{a}F_{k}^{T} + Q_{k}. $$

Similarly, we can construct the measurement covariance estimate by calculating the covariance from our observation equation and adding it to the measurement error covariance $R_{k}$:

15 $$ P^{y}_{k+1} = H_{k+1}P^{f}_{k+1}H_{k+1}^{T} + R_{k}. $$

The Kalman gain is defined analogously to (10):

16 $$ K_{k+1} =P_{k+1}^{f}H_{k+1}^{T} \bigl(P^{y}_{k+1} \bigr)^{-1}. $$

The covariance and the mean estimate of the system are updated through a weighted sum with the Kalman gain:

17 $$\begin{aligned} P_{k+1}^{a} &= (I-K_{k+1}H_{k+1})P_{k+1}^{f} \end{aligned}$$

18 $$\begin{aligned} \hat{x}_{k+1}^{a} &= \hat{x}_{k+1}^{f} + K_{k+1} \bigl(y_{k+1}-\hat{y}_{k+1}^{f} \bigr). \end{aligned}$$

These equations can be interpreted as a predictor–corrector method, where the predictions of the state estimates are $\hat{x}^{f}_{k+1}$ with corresponding uncertainties $P_{k+1}^{f}$ in the *forecast*. The correction, or *analysis*, step linearly interpolates the forecast predictions with observational readings.

In this paper we only consider filters, however *smoothers* are another form of sequential DA that also use observational data from future times $t_{k+2},\ldots, t_{k+l}$ to estimate the state at $t_{k+1}$.

## Nonlinear Data Assimilation Methods

### Nonlinear Filtering

For nonlinear models, the Kalman equations need to be adapted to permit nonlinear mappings in the forward operator and the observation operator:

19 $$\begin{aligned} x_{k+1} &=f(x_{k})+\omega_{k+1} , \quad \omega_{k} \in R^{L}, \end{aligned}$$

20 $$\begin{aligned} y_{k+1} &=h(x_{k+1})+\eta_{k+1}, \quad \eta_{k+1} \in R^{M}. \end{aligned}$$

Our observation operator for voltage data remains linear: $h(x) = Hx=[\mathbf{e_{1}} 0 \dots0] x$, where **ej** is the *j*th elementary basis vector, is a projection onto the voltage component of our system. Note that $h(x)$ is an operator, not to be confused with the inactivation gate in (2). Our nonlinear model update, $f(x)$ in (19), is taken as the forward integration of the dynamical equations between observation times.

Multiple platforms for adapting the Kalman equations exist. The most straightforward approach is the extended Kalman filter (EKF) which uses local linearizations of the nonlinear operators in (19)–(20) and plugs these into the standard Kalman equations. By doing so, one preserves Gaussianity of the state-space. Underlying the data assimilation framework is the goal of understanding the distribution, or statistics of the distribution, of the states of the system given the observations:

21 $$ p(x | y) \propto p(y | x ) p(x). $$

The Gaussianity of the state-space declares the posterior conditional distribution $p(x | y)$ to be a normal distribution by the product of Gaussians being Gaussian, and the statistics of this distribution lead to the Kalman update equations [10]. However, the EKF is really only suitable when the dynamics are nearly linear between observations and can result in divergence of the estimates [11].

**Fig. 1:**
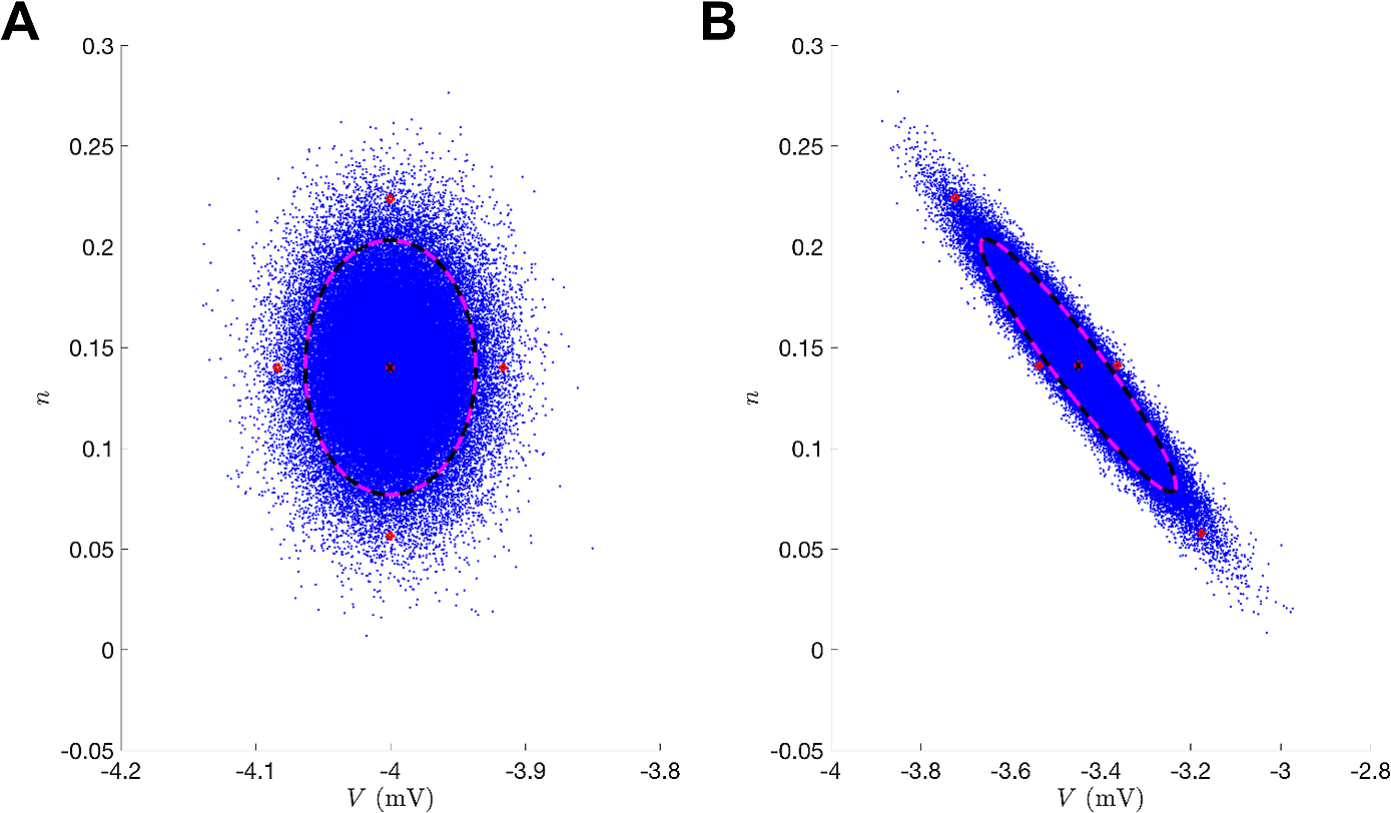
Unscented transformation. (**A**) Initial data where blue corresponds to sampling points from a normal distribution of the $V,n$ state-space and the red circles are the sigma points. Black corresponds to the true uncertainty and mean of the sampled distribution. Magenta corresponds to the statistics of the sigma points. (**B**) Illustrates the forward operator $f(x)$ acting on each element of the left panel where $f(x)$ is the numerical integration of the Morris–Lecar equations (42)–(46) between observation times

Rather than trying to linearize the transformation to preserve Gaussianity, where this distributional assumption is not going to be valid for practical problems anyway, an alternative approach is to preserve the nonlinear transformation and try to estimate the first two moments of transformed state [11]. The Unscented Kalman Filter (UKF) approximates the first two statistics of $p(x_{k} | y_{0}\ldots y_{k} )$ by calculating sample means and variances, which bypasses the need for Gaussian integral products. The UKF uses an ensemble of deterministically selected points in the state-space whose collective mean and covariance are that of the state estimate and its associated covariance at some time. The forward operator $f(x)$ is applied to each of these *sigma points*, and the mean and covariance of the transformed points can then be computed to estimate the nonlinearly transformed mean and covariance. Figure 1 depicts this “unscented” transformation. The sigma points precisely estimate the true statistics both initially (Fig. 1(A)) and after nonlinear mapping (Fig. 1(B)).

In the UKF framework, as with all DA techniques, one is attempting to estimate the states of the system. The standard set of states in conductance-based models includes the voltage, the gating variables, and any intracellular ion concentrations not taken to be stationary. To incorporate parameter estimation, parameters ***θ*** to be estimated are promoted to states whose evolution is governed by the model error random variable:

22 $$ \boldsymbol{\theta}_{k+1}= \boldsymbol{\theta}_{k}+ \omega^{\boldsymbol{\theta}}_{k+1},\quad \omega^{\boldsymbol {\theta}}_{k} \in R^{D}. $$

This is referred to as an “artificial noise evolution model”, as the random disturbances driving deviations in model parameters over time rob them of their time-invariant definition [12, 13]. We found this choice to be appropriate for convergence and as a tuning mechanism. An alternative is to zero out the entries of $Q_{k}$ corresponding to the parameters in what is called a “persistence model” where $\boldsymbol {\theta}_{k+1}= \boldsymbol{\theta}_{k}$ [14]. However, changes in parameters can still occur during the analysis stage.

We declare our augmented state to be comprised of the states in the dynamical system as well as parameters ***θ*** of interest:

23 $$ \mbox{Augmented State:}\quad \mathbf{x} = (V, \mathbf{q}, \boldsymbol{\theta})^{\textrm{T}}, \quad\mathbf{q} \in\mathbb{R}^{L-1}, \boldsymbol{ \theta} \in\mathbb{R}^{D}, $$

where *q* represents the additional states of the system besides the voltage. The filter requires an initial guess of the state $\hat{x}_{0}$ and covariance $P_{xx}$. An implementation of this algorithm is provided as Supplementary Material with the parent function *UKFML.m* and one time step of the algorithm computed in *UKF_Step.m*.

An ensemble of *σ* points are formed and their position and weights are determined by *λ*, which can be chosen to try to match higher moments of the system distribution [11]. Practically, this algorithmic parameter can be chosen to spread the ensemble for $\lambda>0$, shrink the ensemble for $-N< \lambda< 0$, or to have the mean point completely removed from the ensemble by setting it to zero. The ensemble is formed on lines 80-82 of *UKF_Step.m*. The individual weights can be negative, but their cumulative sum is 1.

24 $$\begin{aligned} \begin{aligned} \sigma\mathrm{Points:}\quad X_{j}& = \hat{x}_{k}^{a} \pm \bigl(\sqrt{(N+\lambda)P_{xx}} \bigr)_{j}, \quad j=1,\ldots,2N, \qquad X_{0} = \hat{x}_{k}^{a}, \\ \text{Weights:} \quad W_{j} &= \frac{1}{2 (N+\lambda )},\quad j=1,\ldots,2N, \qquad W_{0} = \frac{\lambda}{N+\lambda}. \end{aligned} \end{aligned}$$

We form our background estimate $\hat{x}_{k+1}^{b}$ by applying our map $f(x)$ to each of the ensemble members

25 $$ \tilde{X}_{j} = f(X_{j}) $$

and then computing the resulting mean:

26 $$ \mbox{Forecast Estimate}: \quad\hat{x}^{b}_{k+1} = \sum _{j=0}^{2N} W_{j}\tilde {X}_{j}. $$

We then propagate the transformed sigma points through the observation operator

27 $$ \tilde{Y}_{j} = h(\tilde{X}_{j}) $$

and compute our predicted observation $\hat{y}_{k+1}^{b}$ from the mapped ensemble:

28 $$ \mbox{Measurement Estimate:}\quad \hat{y}^{b}_{k+1} = \sum _{j=0}^{2N}W_{j}\tilde {Y}_{j}. $$

We compute the background covariance estimate by calculating the variance of the mapped ensemble and adding the process noise $Q_{k}$:

29 $$ \mbox{Background Cov. Est.:} \quad P_{xx}^{f} = \sum _{j=0}^{2N}W_{j} \bigl( \tilde{X}_{j}-\hat{x}_{i+k}^{b} \bigr) \bigl( \tilde{X}_{j}-\hat{x}_{i+k}^{b} \bigr)^{\textrm {T}} + Q_{k} $$

and do the same for the predicted measurement covariance with the addition of $R_{k}$:

30 $$ \mbox{Predicted Meas. Cov.}: \quad P_{yy} = \sum _{j=0}^{2N}W_{j} \bigl( \tilde{Y}_{j}- \hat{y}_{k+1}^{b} \bigr) \bigl( \tilde{Y}_{j}- \hat{y}_{k+1}^{b} \bigr)^{\textrm {T}} + R_{k+1}. $$

The Kalman gain is computed by matrix multiplication of the cross-covariance:

31 $$ \mbox{Cross-Cov.}: \quad P_{xy} =\sum _{j=0}^{2N}W_{j} \bigl( \tilde{X}_{j}- \hat{x}_{k+1}^{b} \bigr) \bigl( \tilde{Y}_{j}- \hat{y}_{k+1}^{b} \bigr)^{\textrm{T}} $$

with the predicted measurement covariance:

32 $$ \mbox{Kalman Gain}: \quad K = P_{xy}P_{yy}^{-1}. $$

When only observing voltage, this step is merely scalar multiplication of a vector. The gain is used in the *analysis*, or update step, to linearly interpolate our background statistics with measurement corrections. The update step for the covariance is

33 $$ P_{xx}^{a} = P_{xx}^{f} - KP_{xy}^{\textrm{T}}, $$

and the mean is updated to interpolate the background estimate with the deviations of the estimated measurement term with the observed data $y_{k+1}$:

34 $$\begin{aligned} \hat{x}_{k+1}^{a} = \hat{x}_{k+1}^{b}+ K \bigl(y_{k+1} - \hat{y}_{k+1}^{b} \bigr). \end{aligned}$$

The analysis step is performed on line 124 of *UKF_Step.m*. Some implementations also include a redistribution of the sigma points about the forecast estimate using the background covariance prior to computing the cross-covariance $P_{xy}$ or the predicted measurement covariance $P_{yy}$ [15]. So, after (29), we redefine $\tilde{X_{j}}$, $\tilde{Y}_{j}$ in (25) as follows:

$$\begin{aligned} \tilde{X_{j}} &= \hat{x}_{k+1}^{b} \pm \bigl( \sqrt{(N+\lambda)P _{xx}} \bigr) _{j}, \quad j=1, \ldots, 2N, \\ \tilde{Y}_{j} &= h(\tilde{X}_{j}). \end{aligned}$$

The above is shown in lines 98–117 in *UKF_Step*. A particularly critical part of using a filter, or any DA method, is choosing the process covariance matrix $Q_{k}$ and the measurement covariance matrix $R_{k}$. The measurement noise may be intuitively based upon knowledge of one’s measuring device, but the model error is practically impossible to know *a priori*. Work has been done to use previous innovations to simultaneously estimate *Q* and *R* during the course of the estimation cycle [16], but this becomes a challenge for systems with low observability (such as is the case when only observing voltage). Rather than estimating the states and parameters simultaneously as with an augmented state-space, one can try to estimate the states and parameters separately. For example, [17] used a shooting method to estimate parameters and the UKF to estimate the states. This study also provided a systematic way to estimate an optimal covariance inflation $Q_{k}$. For high-dimensional systems where computational efficiency is a concern, an implementation which efficiently propagates the square root of the state covariance has been developed [18].

**Fig. 2:**
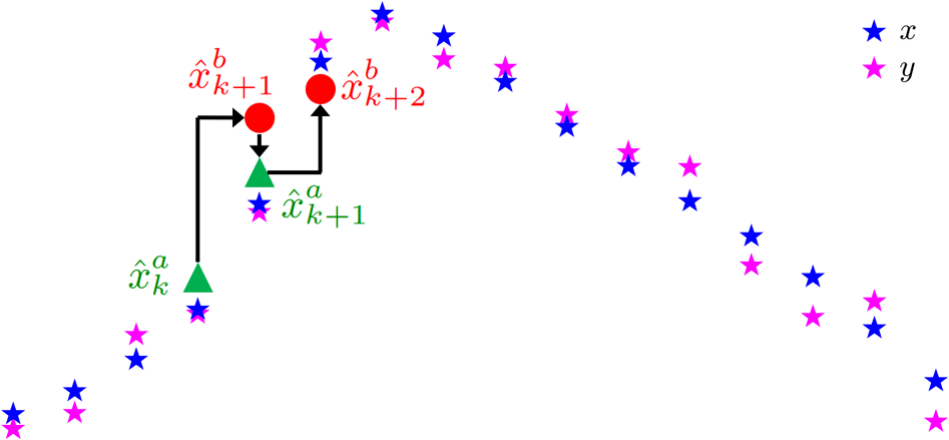
Example of iterative estimation in UKF. The red circles are the result of forward integration through the model using the previous best estimates. The green are the estimates after combining these with observational data. The blue stars depict the true system output (without any noise), and the magenta stars are the noisy observational data with noise generated by (48) and $\varepsilon =0.1$

Figure 2 depicts how the algorithm operates. Between observation times, the previous analysis (or best estimate) point is propagated through the model to come up with the predicted model estimate. The Kalman update step interpolates this point with observations weighted by the Kalman gain.

### Variational Methods

In continuous time, variational methods aim to find minimizers of functionals which represent approximations to the probability distribution of a system conditioned on some observations. As our data is available only in discrete measurements, it is practical to work with a discrete form similar to (7) for nonlinear systems:

35 $$ C(x) = \frac{1}{2}\sum_{k=0}^{N} \bigl\lVert y_{k}-h(x_{k}) \bigr\rVert ^{2}_{R _{k}}+ \frac{1}{2}\sum_{k=0}^{N-1} \bigl\lVert x_{k+1}-f(x_{k}) \bigr\rVert ^{2}_{P ^{b}_{k}} . $$

We assume that the states follow the state-space description in (19)–(20) with $\omega_{k}\sim \mathcal{N}(0,Q)$ and $\eta_{k}\sim\mathcal{N}(0,R)$, where *Q* is our model error covariance matrix and *R* is our measurement error covariance matrix. As an approximation, we impose *Q*, *R* to be diagonal matrices, indicating that there is assumed to be no correlation between errors in other states. Namely, *Q*, contains only the assumed model error variance for each state-space component, and *R* is just the measurement error variance of the voltage observations. These assumptions simplify the cost function to the following:

36 $$ C(x) = \frac{1}{2}\sum_{k=0}^{N}R^{-1} ( y_{k}-V_{k} ) ^{2}+ \frac{1}{2}\sum _{l=1}^{L}\sum_{k=0}^{N-1}Q_{l,l}^{-1} \bigl( x_{l,k+1}-f _{l}(x_{k}) \bigr) ^{2}, $$

where $V_{k} =x_{1,k}$. For the current-clamp data problem in neuroscience, one seeks to minimize equation (36) in what is called the “weak 4D-Var” approach. An example implementation of weak 4D-Var is provided in *w4DvarML.m* in the Supplementary Material. An example of the cost function with which to minimize over is given in the child function *w4dvarobjfun.m*. Each of the $x_{k}$ is mapped by $f(x)$ on line 108. Alternatively, “strong 4D-Var” forces the resulting estimates to be consistent with the model $f(x)$. This can be considered the result of taking $Q \rightarrow\mathbf{0}$, which yields the nonlinearly constrained problem

37 $$ C(x) = \frac{1}{2}\sum_{k=0}^{N}R^{-1} ( y_{k}-V_{k} ) ^{2} $$

such that

38 $$ x_{k+1}= f(x_{k}) , \quad k=0, \ldots, N. $$

The rest of this paper will be focused on the weak case (36), where we can define the argument of the optimization as follows:

39 $$ \mathbf{x} = [x_{1,1} , x _{1,2}, \ldots, x_{1,N}, x_{2,1}, \dots, x_{L,N}, \theta_{1}, \theta_{2}, \dots,\theta_{D}] $$

resulting in an $(N+1)L + D$-dimensional estimation problem. An important aspect of the scalability of this problem is that the Hessian matrix

40 $$ \mathbf{H}_{i,j} = \frac{\partial^{2} C}{\partial x_{i} \partial x_{j}} $$

is sparse. Namely, each state at each discrete time has dependencies based upon the model equations and the chosen numerical integration scheme. At the heart of many gradient-based optimization techniques lies a linear system, involving the Hessian and the gradient $\nabla C(\mathbf{x}_{n})$ of the objective function, that is used to solve for the next candidate point. Specifically, Newton’s method for optimization is

41 $$ \mathbf{x}_{n+1} = \mathbf{x}_{n} - \mathbf{H}^{-1} \nabla C(\mathbf{x}_{n}). $$

Therefore, if $(N+1)L + D$ is large, then providing the sparsity pattern is advantageous when numerical derivative approximations, or functional representations of them, are being used to perform minimization with a derivative-based method. One can calculate these derivatives by hand, symbolic differentiation, or automatic differentiation.

**Fig. 3:**
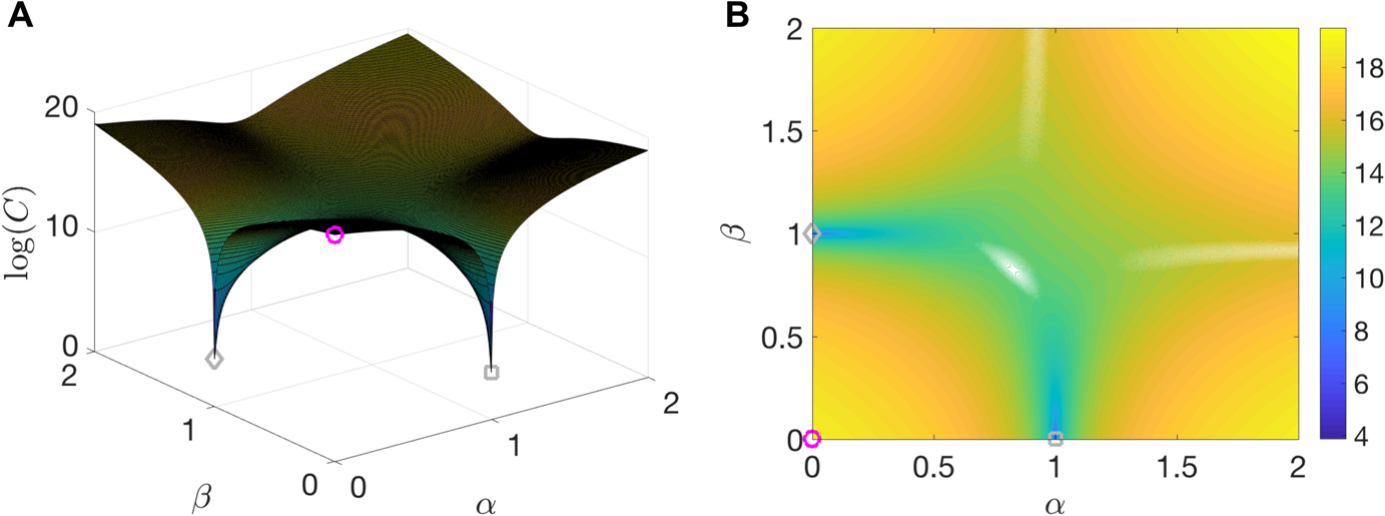
Example cost function for 4D-Var. (**A**) Surface generated by taking the logarithm of $C(\alpha,\beta)$, where $C(\alpha,\beta) = C (\mathbf{x}_{0}(1-\alpha)(1-\beta) + \alpha x_{\textrm{min,d}} +\beta x_{\textrm{min,s}} )$ so that at $\alpha =\beta=0$, $\mathbf{x}=\mathbf{x}_{0}$ (magenta circle), and at $\alpha=1$ and $\beta=0$, $\mathbf{x}=x_{\textrm{min,d}}$ for the deeper minima (gray square), and similarly for the shallower minima (gray diamond). (**B**) Contour plot of the surface shown in (A)

A feature of the most common derivative-based methods is assured convergence to local minima. However, our problem is non-convex due to the model term, which leads to the development of multiple local minima in the optimization surface as depicted in Fig. 3. For the results in this tutorial, we will only utilize local optimization tools, but see Sect. 5 for a brief discussion of some global optimization methods with stochastic search strategies.

## Application to Spiking Regimes of the Morris–Lecar Model

### Twin Experiments

Data assimilation is a framework for the incorporation of system observations into an estimation problem in a systematic fashion. Unfortunately, the methods themselves do not provide a great deal of insight into the tractability of unobserved system components of specific models. There may be a certain level of redundancy in the model equations and degeneracy in the parameter space leading to multiple potential solutions [19]. Also, it may be the case that certain parameters are non-identifiable if, for instance, a parameter can be completely scaled out [20]. Some further work on identifiability is ongoing [21, 22].

Before applying a method to data from a real biological experiment, it is important to test it against simulated data where the ground truth is known. In these experiments, one creates simulated data from a model and then tries to recover the true states and parameters of that model from the simulated data alone.

### Recovery of Bifurcation Structure

In conductance-based models, as well as in real neurons, slight changes in a parameter value can lead to drastically different model output or neuronal behavior. Sudden changes in the topological structure of a dynamical system upon smooth variation of a parameter are called *bifurcations*. Different types of bifurcations lead to different neuronal properties, such as the presence of bistability and subthreshold oscillations [23]. Thus, it is important for a neuronal model to accurately capture the bifurcation dynamics of the cell being modeled [24]. In this paper, we ask whether or not the models estimated through data assimilation match the bifurcation structure of the model that generated the data. This provides a qualitative measure of success or failure for the estimation algorithm. Since bifurcations are an inherently nonlinear phenomenon, our use of topological structure as an assay emphasizes how nonlinear estimation is a fundamentally distinct problem from estimation in linear systems.

### Morris–Lecar Model

**Fig. 4:**
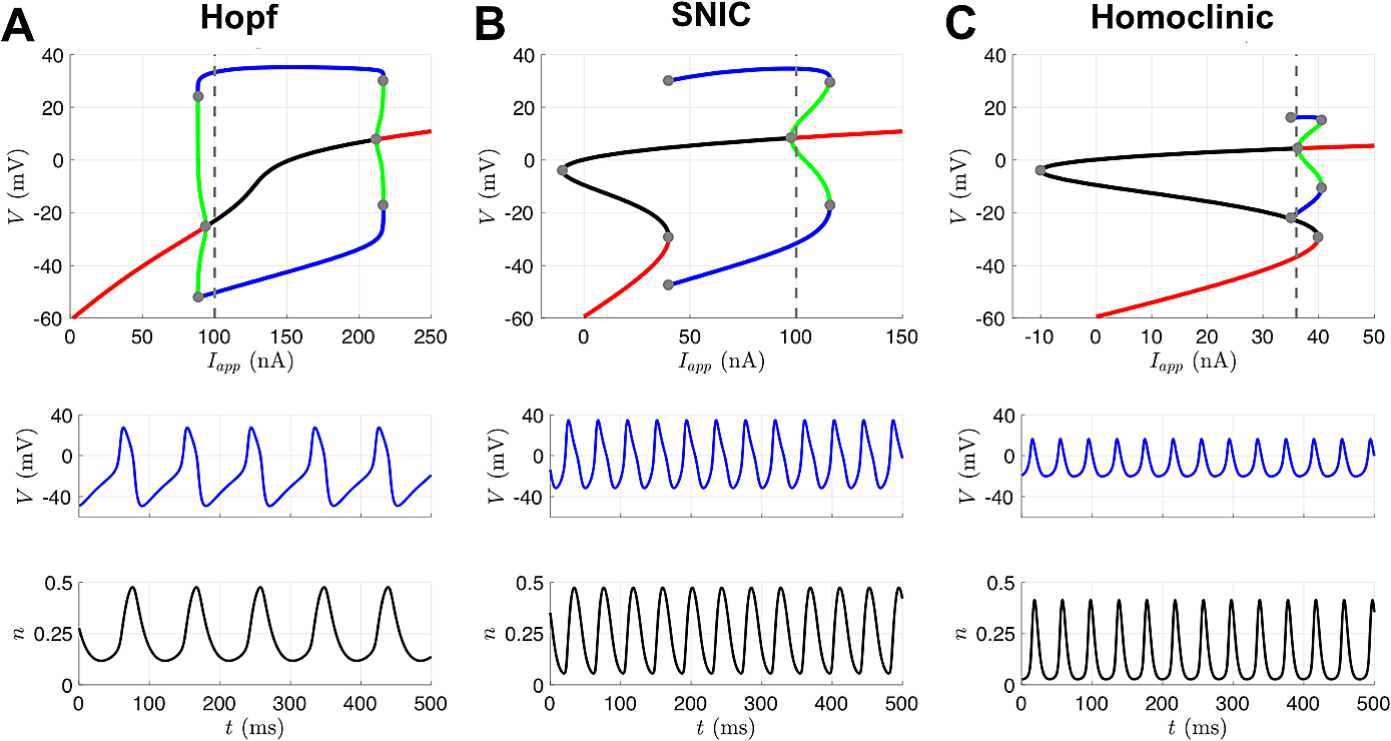
Three different excitability regimes of the Morris–Lecar model. The bifurcation diagrams in the top row depict stable fixed points (red), unstable fixed points (black), stable limit cycles (blue), and unstable limit cycles (green). Gray dots indicate bifurcation points, and the dashed gray lines indicate the value of $I_{ \textrm{app}}$ corresponding to the traces shown for *V* (middle row) and *n* (bottom row). (**A**) As $I_{\textrm{app}}$ is increased from 0 or decreased from 250 nA, the branches of stable fixed points lose stability through subcritical Hopf bifurcation, and unstable limit cycles are born. The branch of stable limit cycles that exists at $I_{\textrm{app}}=100$ nA eventually collides with these unstable limit cycles and is destroyed in a saddle-node of periodic orbits (SNPO) bifurcation as $I_{\textrm{app}}$ is increased or decreased from this value. (**B**) As $I_{\textrm{app}}$ is increased from 0, a branch of stable fixed points is destroyed through saddle-node bifurcation with the branch of unstable fixed points. As $I_{\textrm{app}}$ is decreased from 150 nA, a branch of stable fixed points loses stability through subcritical Hopf bifurcation, and unstable limit cycles are born. The branch of stable limit cycles that exists at $I_{\mathrm{app}}=100$ nA is destroyed through a SNPO bifurcation as $I_{\mathrm{app}}$ is increased and a SNIC bifurcation as $I_{\mathrm{app}}$ is decreased. (**C**) Same as (B), except that the stable limit cycles that exist at $I_{\mathrm{app}}=36$ nA are destroyed through a homoclinic orbit bifurcation as $I_{ \mathrm{app}}$ is decreased

The Morris–Lecar model, first used to describe action potential generation in barnacle muscle fibers, has become a canonical model for studying neuronal excitability [25]. The model includes an inward voltage-dependent calcium current, an outward voltage-dependent potassium current, and a passive leak current. The activation gating variable for the potassium current has dynamics, whereas the calcium current activation gate is assumed to respond instantaneously to changes in voltage. The calcium current is also non-inactivating, resulting in a two-dimensional model. The model exhibits multiple mechanisms of excitability: for different choices of model parameters, different bifurcations from quiescence to repetitive spiking occur as the applied current is increased [23]. Three different bifurcation regimes—Hopf, saddle-node on an invariant circle (SNIC), and homoclinic—are depicted in Fig. 4 and correspond to the parameter sets in Table 1. For a given applied current in the region where a stable limit cycle (corresponding to repetitive spiking) exists, each regime displays a distinct firing frequency and action potential shape.

**Table 1:** Morris–Lecar parameter values. For all simulations, $C=20$, $E_{\textrm{Ca}}=120$, $E_{\textrm{K}}=-84$, and $E_{ \textrm{L}}=-60$. For the Hopf and SNIC regime, $I_{\textrm{app}}=100$; for the homoclinic regime, $I_{\textrm{app}}=36$

	Hopf	SNIC	Homoclinic
*ϕ*	0.04	0.067	0.23
$g_{\textrm{Ca}}$	4	4	4
$V_{3}$	2	12	12
$V_{4}$	30	17.4	17.4
$g_{\textrm{K}}$	8	8	8
$g_{\textrm{L}}$	2	2	2
$V_{1}$	−1.2	−1.2	−1.2
$V_{2}$	18	18	18

The equations for the Morris–Lecar model are as follows:

42 $$\begin{aligned} C_{m} \frac{dV}{dt} &=I_{\textrm{app}} -g_{\textrm{L}} (V-E _{\textrm{L}}) - g_{\textrm{K}}n(V-E_{\textrm{K}}) \\ &\quad{}-g_{\textrm{Ca}}m_{\infty}(V) (V-E_{\textrm{Ca}}) \\ &=f^{\star}_{V}(V,n;\boldsymbol{\theta}), \end{aligned}$$

43 $$\begin{aligned} \frac{dn}{dt} & = \phi \bigl(n_{\infty}(V)-n \bigr)/ \tau_{n}(V) = f^{\star}_{n}(V,n;\boldsymbol{\theta}), \end{aligned}$$

with

44 $$\begin{aligned} m_{\infty} &= \frac{1}{2} \bigl[1+\tanh \bigl((V-V_{1})/ V_{2} \bigr) \bigr], \end{aligned}$$

45 $$\begin{aligned} \tau_{n} &= 1/\cosh \bigl((V-V_{3})/2 V_{4} \bigr), \end{aligned}$$

46 $$\begin{aligned} n_{\infty} &= \frac{1}{2} \bigl[1+\tanh \bigl((V-V_{3})/ V_{4} \bigr) \bigr]. \end{aligned}$$

The eight parameters that we will attempt to estimate from data are $g_{\textrm{L}}$, $g_{\textrm{K}}$, $g_{\textrm{{Ca}}}$, *ϕ*, $V_{1}$, $V_{2}$, $V_{3}$, and $V_{4}$. We are interested in whether the estimated parameters yield a model with the desired mechanism of excitability. Specifically, we will conduct twin experiments where the observed data is produced by a model with parameters in a certain bifurcation regime, but the data assimilation algorithm is initialized with parameter guesses corresponding to a different bifurcation regime. We then assess whether or not a model with the set of estimated parameters undergoes the same bifurcations as the model that produced the observed data. This approach provides an additional qualitative measure of estimation accuracy, beyond simply comparing the values of the true and estimated parameters.

### Results with UKF

The UKF was tested on the Morris–Lecar model in an effort to simultaneously estimate *V* and *n* along with the eight parameters in Table 1. Data was generated via a modified Euler scheme at observation points every 0.1 ms, where we take the step-size Δ*t* as 0.1 as well:

47 $$\begin{aligned} \begin{aligned} \tilde{x}_{k+1} &= x_{k} + \Delta t f^{\star}(t_{k},x_{k}), \\ {x}_{k+1} &= x_{k} + \frac{\Delta t}{2} \bigl( f^{\star}(t_{k},x_{k}) + f^{\star}(t_{k+1}, \tilde{x}_{k+1}) \bigr) \\ &=f(x_{k}). \end{aligned} \end{aligned}$$

The UKF is a particularly powerful tool when a lot of data is available; the computational complexity in time is effectively the same as the numerical scheme of choice, whereas the additional operations at each time point are $O((L+D)^{3})$ [26]. $f(x)$ in (19) is taken to be the Morris–Lecar equations (42)–(43), acting as $f^{\star}(t_{k},x_{k})$, integrated forward via modified Euler (47), and is given on line 126 of *UKFML.m*. The function *fXaug.m*, provided in the Supplementary Material, represents our augmented vector field. Our observational operator *H* is displayed on line 136 of *UKFML.m*. To reiterate, the states to be estimated in the Morris–Lecar model are the voltage and the potassium gating variable. The eight additional parameters are promoted to the members of state-space with trivial dynamics resulting in a ten-dimensional estimation problem.

These examples were run using 20 seconds of data which is 200,001 time points. During this time window, the Hopf, SNIC, and homoclinic models fire 220, 477, and 491 spikes, respectively. Such a computation for a ten-dimensional model takes only a few minutes on a laptop computer. *R* can be set to 0 when one believes the observed signal to be completely noiseless, but even then it is commonly left as a small number to try to mitigate the development of singularities in the predicted measurement covariance. We set our observed voltage to be the simulated output using modified Euler with additive white noise at each time point:

48 $$ V_{\textrm{obs}}(t)= V_{\textrm{true}}(t)+\eta(t), $$

where $\eta\sim\mathcal{N}(0,(\varepsilon \sigma_{\textrm{true}})^{2})$ is a normal random variable whose variance is equal to the square of the standard deviation of the signal scaled by a factor *ε*, which is kept fixed at 0.01 for these simulations. *R* is taken as the variance of *η*. The initial covariance of the system is $\alpha_{I}I$, where *I* is the identity matrix and $\alpha_{I}$ is 0.001. The initial guess for *n* is taken to be 0. *Q* is fixed in time as a diagonal matrix with diagonal 10^−7^ $[ \max(V_{\textrm{obs}})-\min(V_{\textrm{obs}}), 1,\lvert \boldsymbol{\theta_{0}}\rvert ] $, where $\boldsymbol{\theta_{0}}$ represents our initial parameter guesses. We set $\lambda=5$; however, this parameter was not especially influential for the results of these runs, as discussed further below. These initializations are displayed in the body of the parent function *UKFML.m*.

**Fig. 5:**
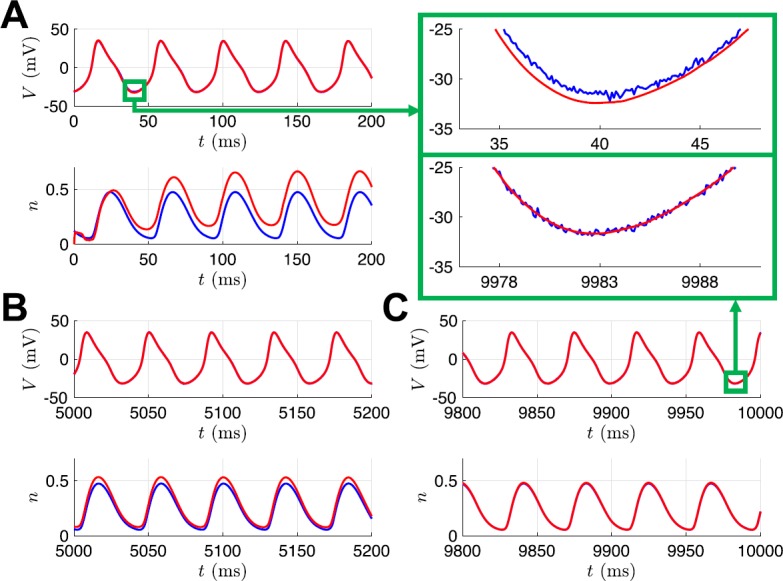
State estimates for UKF. This example corresponds to initializing with parameters from the HOPF regime and attempting to correctly estimate those of the SNIC regime. The noisy observed voltage *V* and true unobserved gating variable *n* are shown in blue, and their UKF estimates are shown in red

**Fig. 6:**
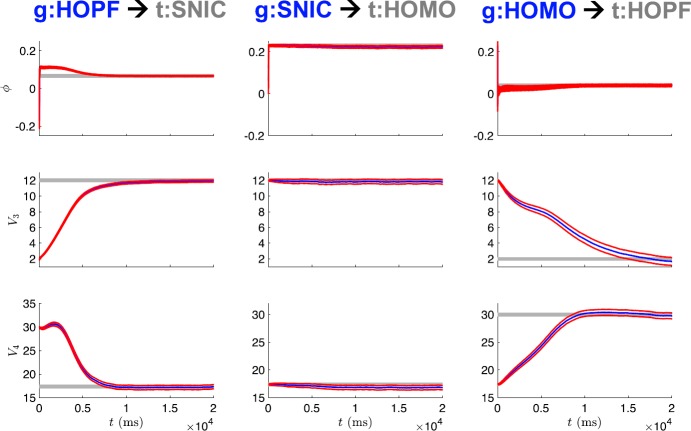
Parameter estimates for UKF. This example corresponds to initializing with parameters from the HOPF, SNIC, and HOMO regimes and attempting to correctly estimate those of the SNIC, HOMO, and HOPF regimes (left to right column, respectively). The blue curves are the estimates from the UKF, with ±2 standard deviations from the mean (based on the filter estimated covariance) shown in red. The gray lines indicate the true parameter values

Figure 5 shows the state estimation results when the observed voltage is from the SNIC regime, but the UKF is initialized with parameter guess corresponding to the Hopf regime. Initially, the state estimate for *n* and its true, unobserved dynamics have great disparity. As the observations are assimilated over the estimation window, the states and model parameters adjust to produce estimates which better replicate the observed, and unobserved, system components. In this way, information from the observations is transferred to the model. The evolution of the parameter estimates for this case is shown in the first column of Fig. 6, with *ϕ*, $V_{3}$, and $V_{4}$ all converging to close to their true values after 10 seconds of observations. The only difference in parameter values between the SNIC and homoclinic regimes is the value of the parameter *ϕ*. The second column of Fig. 6 shows that when the observed data is from the homoclinic regime but the initial parameter guesses are from the SNIC regime, the estimates of $V_{3}$ and $V_{4}$ remain mostly constant near their original (and correct) values, whereas the estimate of *ϕ* quickly converges to its new true value. Finally, the third column of Fig. 6 shows that all three parameter estimates evolve to near their true values when the UKF is presented with data from the Hopf regime but initial parameter estimates from the homoclinic regime.

**Fig. 7:**
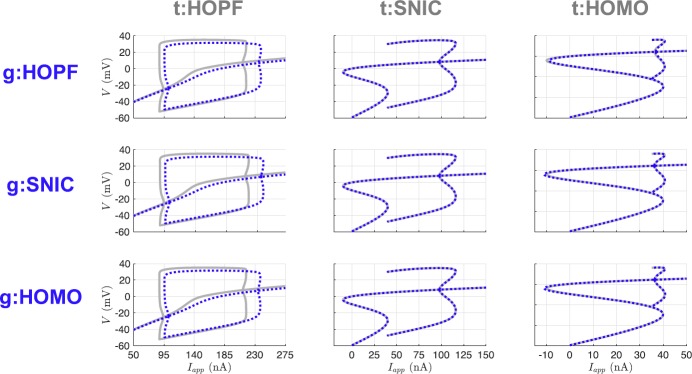
Bifurcation diagrams for UKF twin experiments. The gray lines correspond to the true diagrams, and the blue dotted lines correspond to the diagrams produced from the estimated parameters in Table 2

Table 2 shows the parameter estimates at the end of the estimation window for all of the nine possible twin experiments. Promisingly, a common feature of the results is the near recovery of the true value of each of the parameters. However, the estimated parameter values alone do not necessarily tell us about the dynamics of the inferred model. To assess the inferred models, we generate bifurcation diagrams using the estimated parameters and compare them to the bifurcation diagrams for the parameters that produced the observed data. Figure 7 shows that the SNIC and homoclinic bifurcation diagrams were recovered quite exactly. The Hopf structure was consistently recovered, but with shifted regions of spiking and quiescence and minor differences in spike amplitude.

**Table 2:** UKF parameter estimates at end of estimation window, with observed data from bifurcation regime ‘t’ and initial parameter guesses corresponding to bifurcation regime ‘g’

	t:HOPF			t:SNIC			t:HOMO		
	g:HOPF	g:SNIC	g:HOMO	g:HOPF	g:SNIC	g:HOMO	g:HOPF	g:SNIC	g:HOMO
*ϕ*	0.040	0.40	0.040	0.067	0.040	0.067	0.237	0.224	0.224
$g_{\textrm{Ca}}$	4.017	4.019	4.025	4.001	4.000	4.001	4.112	3.874	3.877
$V_{3}$	1.612	1.762	1.660	11.931	11.937	11.912	11.751	11.784	11.772
$V_{4}$	29.646	29.832	29.771	17.343	17.337	17.342	17.739	16.806	16.815
$g_{\textrm{K}}$	7.895	7.926	7.892	7.970	7.971	7.958	7.929	7.854	7.850
$g_{\textrm{L}}$	2.032	2.027	2.033	2.003	2.004	2.003	2.025	1.967	1.968
$V_{1}$	−1.199	−1.195	−1.189	−1.193	−1.193	−1.190	−1.064	−1.346	−1.341
$V_{2}$	18.045	18.053	18.067	17.991	17.991	17.991	18.179	17.734	17.740

**Fig. 8:**
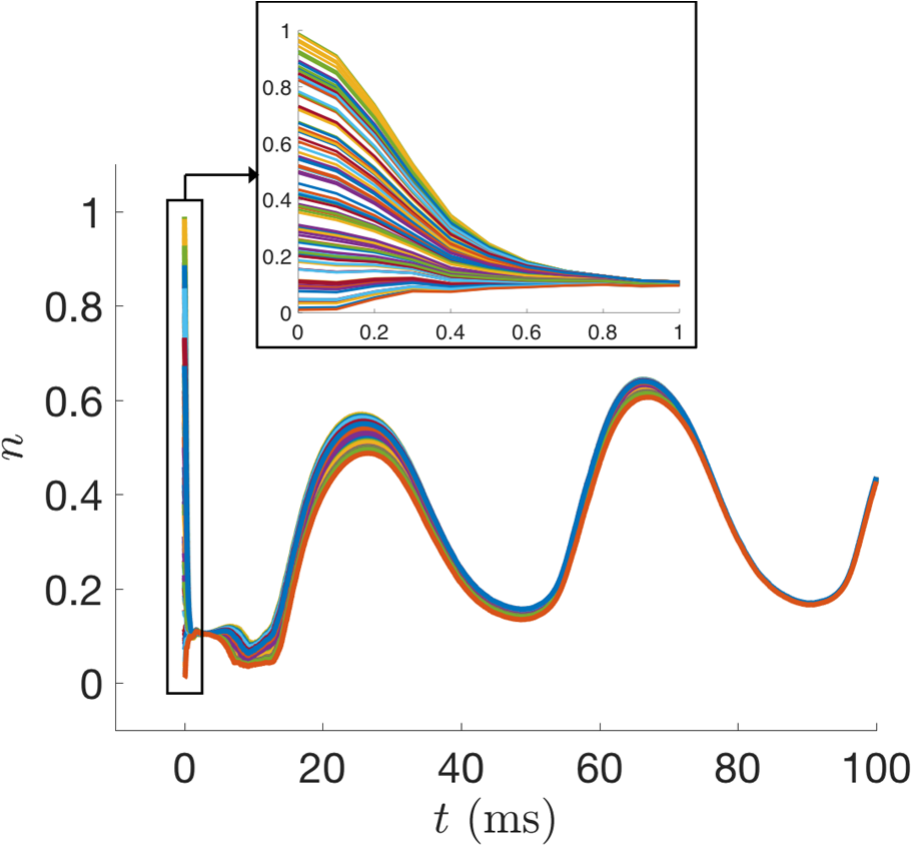
UKF state estimates of *n* for the Morris–Lecar model with 100 different initial guesses of the state sampled from $\mathcal {U}(0,1)$, with all other parameters held fixed

**Fig. 9:**
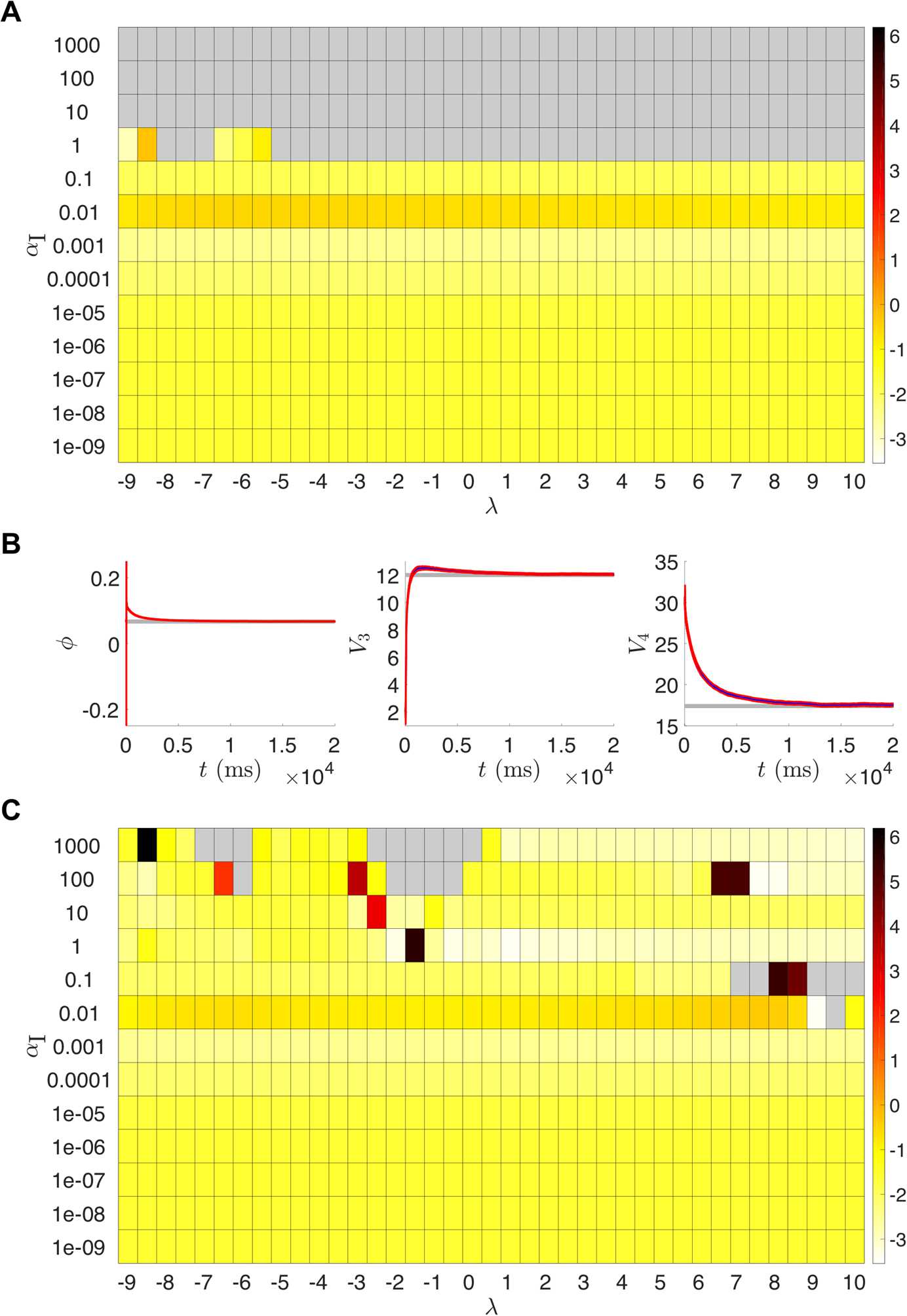
(**A**) UKF results from runs of the t:SNIC/g:HOPF twin experiment for various parameter combinations of *λ* and $\alpha _{I}$. The color scale represents the root mean squared error of the final parameter values at $T=200\text{,}001$ from the parameters of the SNIC bifurcation regime. Gray indicates the filter failed outright before reaching the end of the estimation window. (**B**) Parameter estimates over time for the run with $\lambda=5, \alpha_{I}=0.1$. The parameters (especially *ϕ* and $V_{3}$) approach their true values more quickly than corresponding runs with smaller initial covariances; see column 1 of Fig. 6 for parameter estimates with $\lambda=5, \alpha_{I}=0.001$. **C:** Same as (A), but with a modification to the numerical integration scheme that restricts the gating variable *n* to remain within its biophysical range of 0 to 1

To check the consistency of our estimation, we set 100 initial guesses for *n* across its dynamical range as samples from $\mathcal{U}(0,1)$. Figure 8 shows that the state estimates for *n* across these initializations quickly approached very similar trajectories. We confirmed that after the estimation cycle was over, the parameter estimates for all 100 initializations were essentially identical to the values shown in Table 2. In this paper, we always initialized the UKF with initial parameter values corresponding to the various bifurcation regimes and did not explore the performance for randomly selected initial parameter guesses. For initial parameter guesses that are too far from the true values, it is possible that the filter would converge to incorrect parameter values or fail outright before reaching the end of the estimation window. Additionally, we investigated the choices of certain algorithmic parameters for the UKF, namely *λ* and $\alpha_{I}$. Figure 9(A) shows suitable ranges of these parameters, with the color indicating the root mean squared error of the parameters at the end of the cycle compared to their true values. We found this behavior to be preserved across our nine twin experiment scenarios. Notably, this shows that our results in Table 2 were generated using an initial covariance $\alpha_{I}=0.001$ that was smaller than necessary. By increasing the initial variability, the estimated system can converge to the true dynamics more quickly, as shown for $\alpha_{I}=0.1$ in Fig. 9(B). The value of *λ* does not have a large impact on these results, except for when $\alpha_{I}=1$. Here the filter fails before completing the estimation cycle, except for a few cases where *λ* is small enough to effectively shrink the ensemble spread and compensate for the large initial covariance. For example, with $\lambda=-9$, we have $N-9=1$ and, therefore, the ensemble spread in (24) is simply $X_{j}=\hat{x}^{a}_{k}\pm\sqrt{P_{xx}}$. For even larger initial covariances ($\alpha_{I}>1$), the filter fails regardless of the value of *λ*. We noticed that in many of the cases that failed, the parameter estimate for *ϕ* was becoming negative (which is unrealistic for a rate) or quite large ($\phi>1$), and that the state estimate for *n* was going outside of its biophysical range of 0 to 1. When the gating variable extends outside of its dynamical range it can skew the estimated statistics and the filter may be unable to recover. The standard UKF framework does not provide a natural way of incorporating bounds on parameter estimates, and we do not apply any for the results presented here. However, we did find that we can modify our numerical integration scheme to prevent the filter from failing in many of these cases, as shown in Fig. 9(C). Specifically, if *n* becomes negative or exceeds 1 after the update step, then artificially setting *n* to 0 or 1 in the modified Euler method (47) before proceeding can enable the filter to reach the end of the estimation window and yield reasonable parameter estimates.

### Results with 4D-Var

The following results illustrate the use of weak 4D-Var. One can minimize the cost function (36) using a favorite choice of optimization routine. For the following examples, we will consider a local optimizer by using interior point optimization with MATLAB’s built-in solver *fmincon*. At the heart of the solver is a Newton-step which uses information about the Hessian, or a conjugate gradient step using gradient information [27–29]. The input we are optimizing over conceptually takes the form of

49 $$ \mathbf{x} = [V_{0}, V_{1}, \dots, V_{N}, n_{0}, n_{1}, \dots, n_{N}, \theta_{1}, \theta_{2}, \dots,\theta_{D}] $$

resulting in an $(N+1)L + D$-dimensional estimation problem where $L=2$. There are computational limitations with memory storage and the time required to sufficiently solve the optimization problem to a suitable tolerance for reasonable parameter estimates. Therefore, we cannot be cavalier with using as much data with 4D-Var as we did with the UKF, as that would result in a $(200\text{,}001)2 + 8 = 400\text{,}010$ dimensional problem. Using Newton’s method (41) on this problem would involve inverting a Hessian matrix of size $(400\text{,}010)^{2}$, which according to a rough calculation would require over 1 TB of RAM. Initialization of the optimization is shown on line 71 of *w4DVarML.m*.

The estimated parameters are given in Table 3. These results were run using $N=2001$ time points. To simplify the search space, the parameter estimates were constrained between the bounds listed in Table 4. These ranges were chosen to ensure that the maximal conductances, the rate *ϕ*, and the activation curve slope $V_{2}$ all remain positive. We found that running 4D-Var with even looser bounds (Table A1) yielded less accurate parameter estimates (Tables A2 and A3). The white noise perturbations for the 4D-Var trials were the same as those from the UKF examples. Initial guesses for the states at each time point are required. For these trials, *V* is initialized as $V_{\textrm{obs}}$, and *n* is initialized as the result of integration of its dynamics forced with $V_{\textrm{obs}}$ using the initial guesses for the parameters, i.e., $n = \int f_{n}(V_{\textrm{obs}},n; \boldsymbol{\theta_{0})}$. The initial guesses are generated beginning on line 38 of *w4DvarML.m*. We impose that $Q^{-1}$ in (36) is a diagonal matrix with entries $\alpha_{Q} [1, 100^{2}]$ to balance the dynamical variance of *V* and *n*. The scaling factor $\alpha_{Q}$ represents the relative weight of the model term compared to the measurement term. Based on preliminary tuning experiments, we set $\alpha_{Q}=100$ for the results presented.

**Table 3:** 4D-Var parameter estimates at the end of the optimization for each bifurcation regime. The parameter bounds in Table 4 were used for these trials. Hessian information was not provided to the optimizer

	t:HOPF			t:SNIC			t:HOMO		
	g:HOPF	g:SNIC	g:HOMO	g:HOPF	g:SNIC	g:HOMO	g:HOPF	g:SNIC	g:HOMO
*ϕ*	0.040	0.037	0.039	0.069	0.067	0.066	0.414	0.218	0.230
$g_{\textrm{Ca}}$	4.000	3.890	3.976	4.024	4.000	4.045	9.037	3.813	3.999
$V_{3}$	2.000	3.404	3.241	12.695	12.000	12.076	7.458	13.022	12.004
$V_{4}$	30.000	29.085	30.122	18.759	17.400	16.990	28.365	17.165	17.403
$g_{\textrm{K}}$	8.000	8.386	8.287	8.284	8.000	8.009	9.817	8.472	8.002
$g_{\textrm{L}}$	2.000	2.016	2.021	1.930	2.000	2.071	3.140	1.941	2.000
$V_{1}$	−1.200	−1.335	−1.250	−1.078	−1.200	−1.179	2.872	−1.419	−1.202
$V_{2}$	18.000	17.619	17.911	18.091	18.000	18.162	24.769	17.712	18.000

**Table 4:** Bounds used during 4D-Var estimation for the results shown in Tables 3 and A4

	Lower bound	Upper bound
*ϕ*	0	1
$g_{\textrm{Ca}}$	0	10
$V_{3}$	−20	20
$V_{4}$	0.1	35
$g_{\textrm{K}}$	0	10
$g_{\textrm{L}}$	0	5
$V_{1}$	−10	20
$V_{2}$	0.1	35

**Fig. 10:**
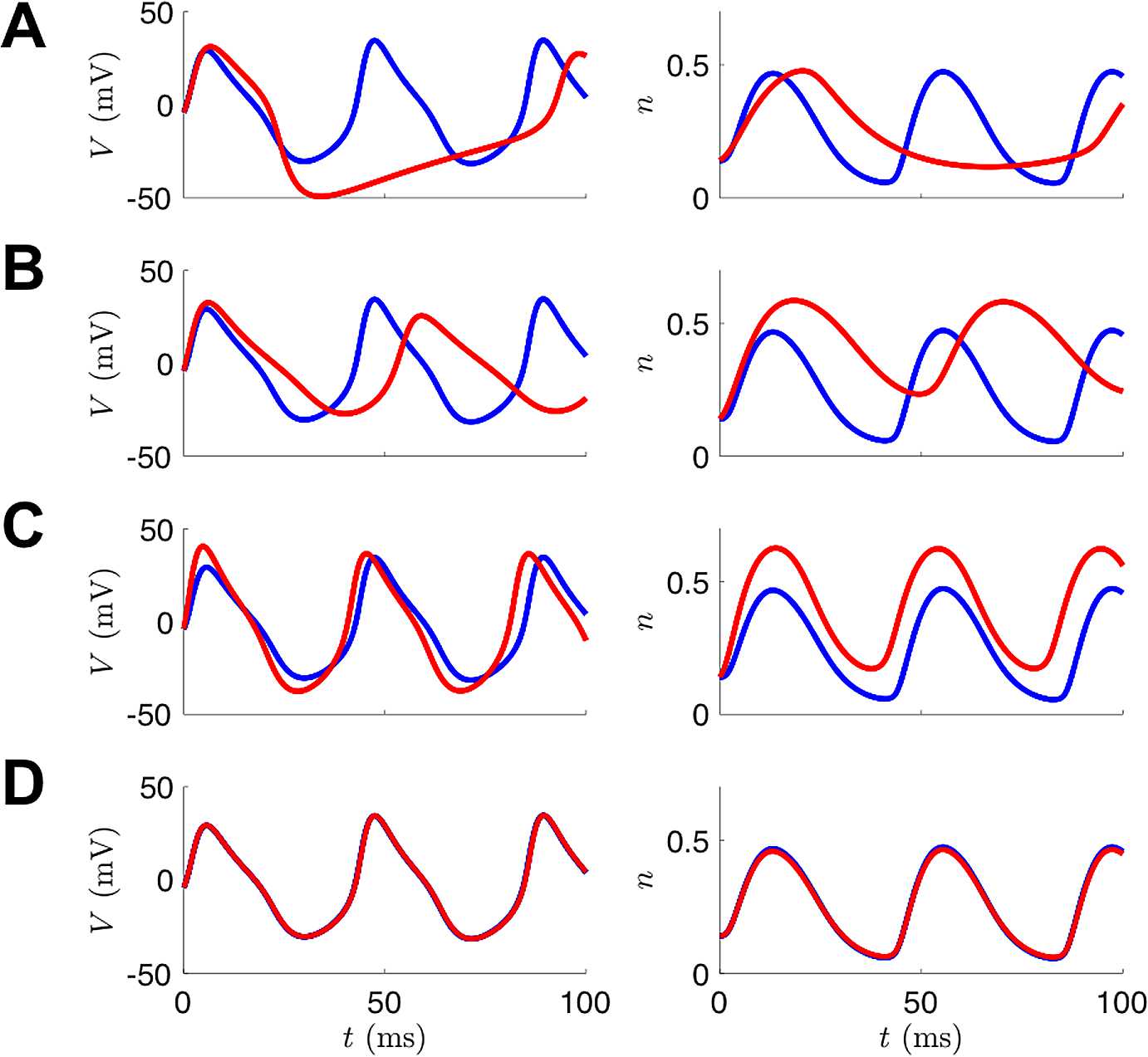
Example of 4D-Var assimilation initializing with parameters from the Hopf regime but observational data from the SNIC regime. The blue traces are noiseless versions of the observed voltage data (left column) or the unobserved variable *n* (right column) from the model that produced the data. The red traces are the result of integrating the model with the estimated parameter sets at various points during the course of the optimization. (**A**) Initial parameter guesses. (**B**) Parameter values after 100 iterations. **C:** Parameter values after 1000 iterations. **D:** Parameter values after 30,000 iterations (corresponds to t:SNIC/g:HOPF column of Table 3)

**Fig. 11:**
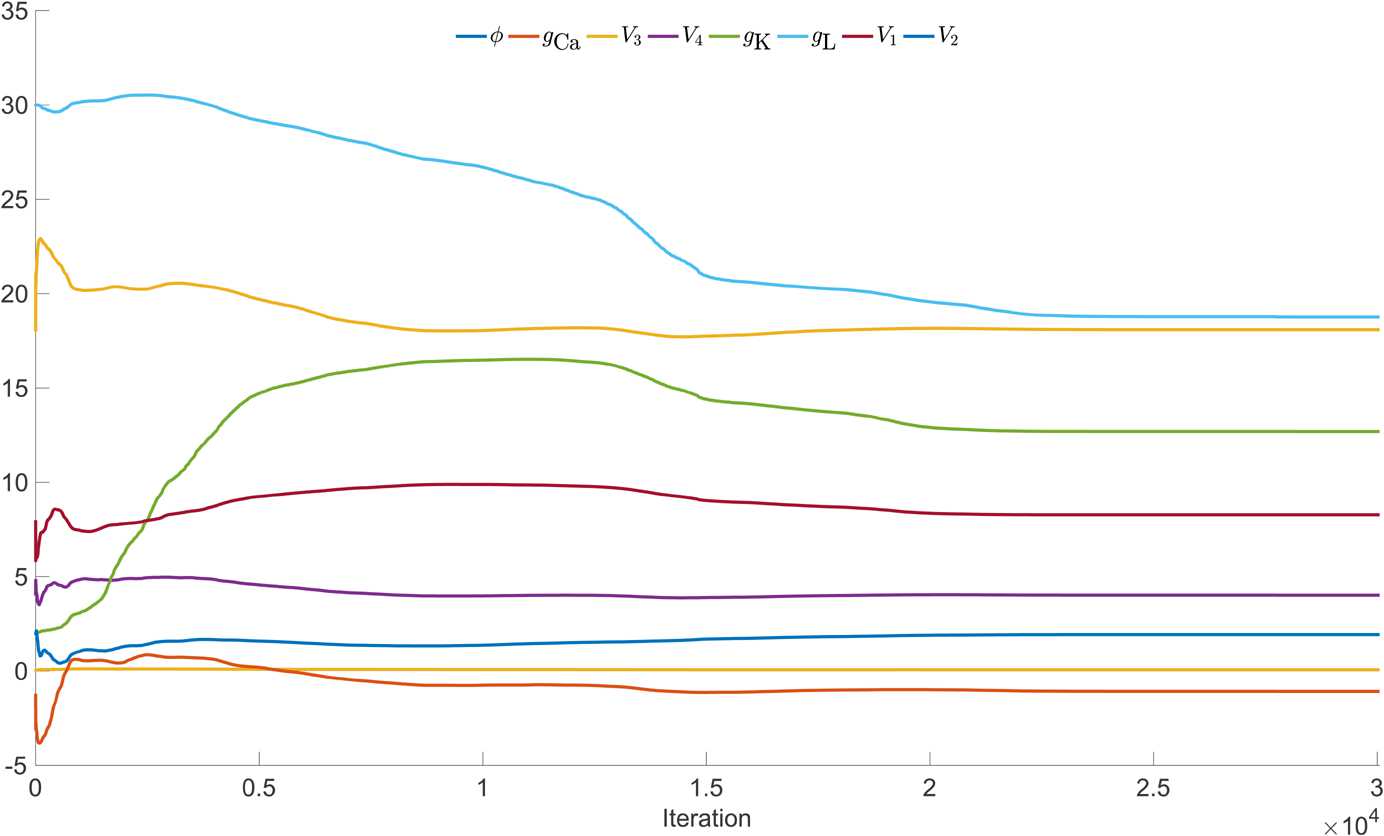
Example parameter estimation with 4D-Var initializing with Hopf parameter regime and estimating parameters of SNIC regime

Figure 10 depicts the states produced by integrating the model with the estimated parameters across different iterations within the interior-point optimization. Over iteration cycles, the geometry of spikes as well as the spike time alignments eventually coincide with the noiseless data $V_{\textrm{true}}$. Figure 11 shows the evolution of the parameters across the entire estimation cycle. For the UKF, the “plateauing” effect of the parameter estimates seen in Fig. 6 indicates confidence that they are conforming to being constant in time. With 4D-Var, and in a limiting sense of the UKF, the plateauing effect indicates the parameters are settling into a local minimum of the cost function.

**Fig. 12:**
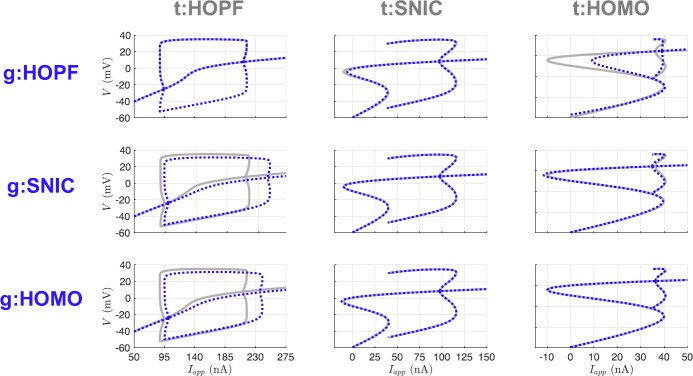
Bifurcation diagrams for 4D-Var twin experiments. The gray lines correspond to the true diagrams, and the blue dotted lines correspond to the diagrams produced from the estimated parameters in Table 3

In Fig. 12 we show the bifurcation diagrams of the estimated models from our 4D-Var trials. Notice, and shown explicitly in Table 3, when initializing with the true parameters, the correct model parameters are recovered as our optimization routine is confidently within the basin of attraction of the global minimum. In the UKF, comparatively, there is no sense of stopping at a local minimum. Parameter estimates may still fluctuate even when starting from their true values, unless the variances of the state components fall to very low values and the covariance $Q_{k}$ can be tuned to have a baseline variability in the system. The parameter sets for the SNIC and homoclinic bifurcation regimes only deviate in the *ϕ* parameter, and so our optimization had great success estimating one from the other. The kinetic parameters ($V_{3}$ and $V_{4}$) for the Hopf regime deviate quite a bit from the SNIC or homoclinic. Still, the recovered bifurcation structures from estimated parameters associated with trials involving HOPF remained consistent with the true structure.

**Fig. 13:**
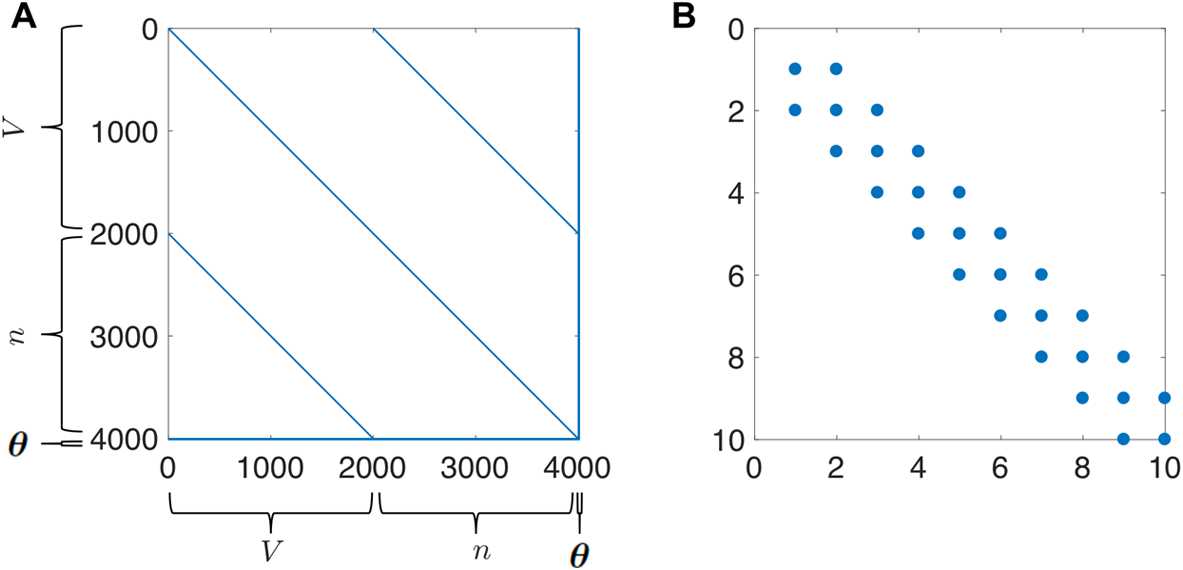
(**A**) Sparsity pattern for the Hessian of the cost function for the Morris–Lecar equations for $N+1=2001$ time points. The final eight rows (and symmetrically the last eight columns) depict how the states at each time depend upon the parameters. (**B**) The top left corner of the Hessian shown in (A)

A drawback of the results shown in Table 3 is that for the default tolerances in *fmincon*, some runs took more than two days to complete on a dedicated core. Figure 11 shows that the optimal solution had essentially been found after 22,000 iterations; however, the optimizer kept running for several thousand more iterations before the convergence tolerances were met. Rather than attempting to speed up these computations by adjusting the algorithmic parameters associated with this solver for this specific problem, we decided to try to exploit the dynamic structure of the model equations using automatic differentiation (AD). AD deconstructs derivatives of the objective function into elementary functions and operations through the chain rule. We used the MATLAB AD tool ADiGator, which performs source transformation via operator overloading and has scripts available for simple integration with various optimization tools, including *fmincon* [30]. For the same problem scenario and algorithmic parameters, we additionally passed in the generated gradient and Hessian functions to the solver. For this problem, the Hessian structure is shown in Fig. 13. Note that we are using a very simple scheme in the modified Euler method (47) to perform numerical integration between observation points, and the states at $k+1$ only have dependencies upon those at *k* and on the parameters. Higher order methods, including implicit methods, can be employed naturally since the system is being estimated simultaneously. A tutorial specific to collocation methods for optimization has been developed [31].

**Fig. 14:**
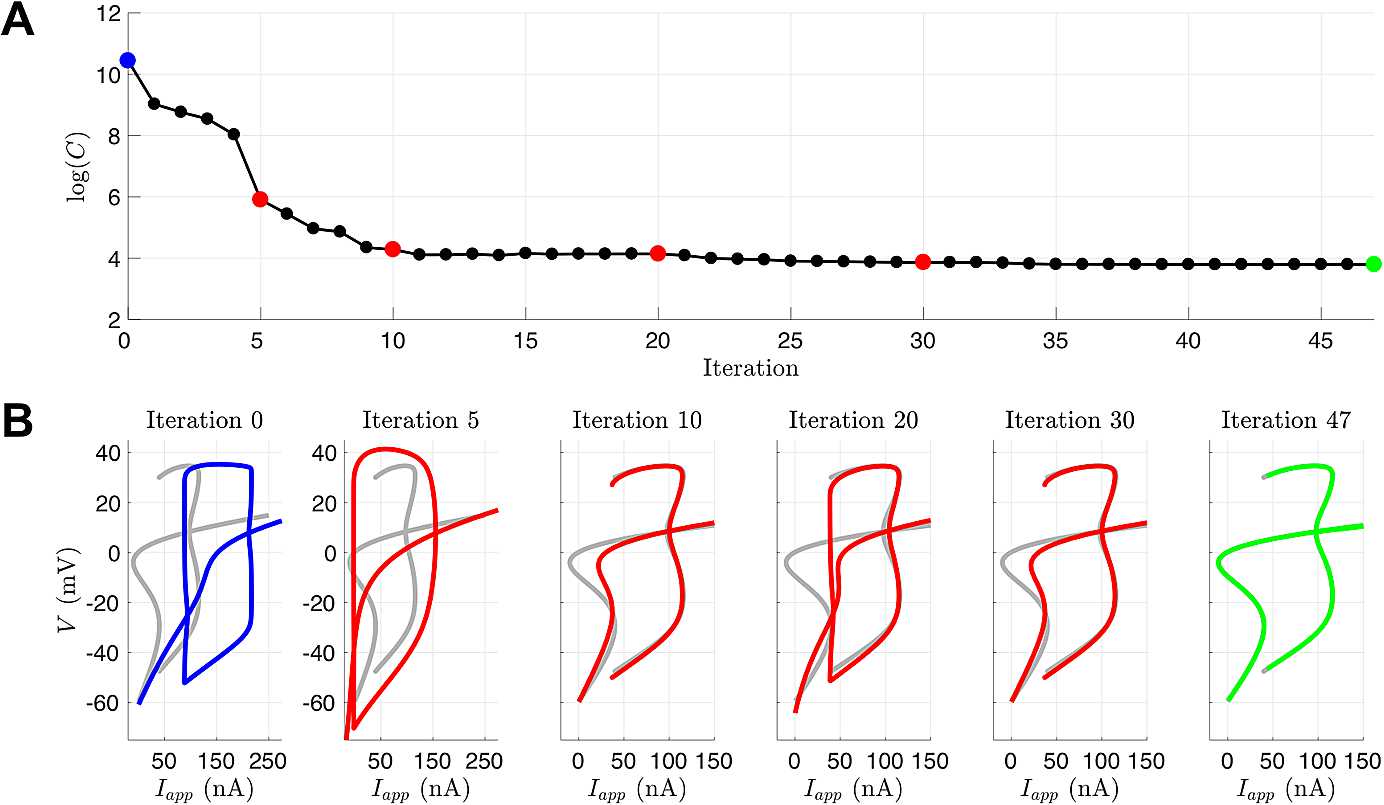
(**A**) Logarithm of the value of the cost function for a twin experiment initialized with parameters from the Hopf regime but observational data from the SNIC regime. The iterates were generated from *fmincon* with provided Hessian and gradient functions. (**B**) Bifurcation diagrams produced from parameter estimates for selected iterations. The blue is the initial bifurcation structure, the gray is the true bifurcation structure for the parameters that generated the observed data, the red is the bifurcation structure of the iterates, and the green is the bifurcation structure of the optimal point determined by *fmincon*

The results are shown in Table A4. Each twin experiment scenario took, at most, a few minutes on a dedicated core. These trials converged to the optimal solution in much fewer iterations than the trials without using the Hessian. Since convergence was achieved within a few dozen iterations, we decided to inspect how the bifurcation structure of the estimated model evolved throughout the process for the case of HOPF to SNIC. Figure 14 shows that by Iteration 10, the objective function value has decreased greatly, and parameters that produce a qualitatively correct bifurcation structure have been found. The optimization continues for another 37 iterations and explores other parts of parameter space that do not yield the correct bifurcation structure before converging very close to the true parameter values.

Again, these results, at best, can reflect only locally optimal solutions of the optimization manifold. The 4D-Var framework has been applied to neuroscience using a more systematic approach to finding the global optimum. In [32], a population of initial states **x** is optimized in parallel with an outer loop that incorporates an *annealing* algorithm. The annealing parameter relates the weights of the two summations in (36), and the iteration proceeds by increasing the weight given to the model error compared to the measurement error.

We also wished to understand more about the sensitivity of this problem to initial conditions. We initialized the system with the voltage states as those of the observation, the parameters as those of the initializing guess bifurcation regime, and the gating variable $[n_{0}, n_{1},\dots n_{N}]$ to be i.i.d. from $\mathcal{U}(0,1)$. The results confirm our suspicions that multiple local minima exist. For 100 different initializations of *n*, for the problem of going from SNIC to HOPF, 63 were found to fall into a deeper minima, yielding better estimates and a smaller objective function value, while 16 fell into a shallower minima, and the rest into three different even shallower minima. While one cannot truly visualize high-dimensional manifolds, one can try to visualize a subset of the surface. Figure 3 shows the surface that arises from evaluating the objective function on a linear combination of the two deepest minima and an initial condition $\mathbf{x}_{0}$, which eventually landed in the shallower of the two minima as points in 4010-dimensional space.

## Application to Bursting Regimes of the Morris–Lecar Model

Many types of neurons display burst firing, consisting of groups of spikes separated by periods of quiescence. Bursting arises from the interplay of fast currents that generate spiking and slow currents that modulate the spiking activity. The Morris–Lecar model can be modified to exhibit bursting by including a calcium-gated potassium (K$_{ \textrm{Ca}}$) current that depends on slow intracellular calcium dynamics [33]:

50 $$\begin{aligned} C_{m} \frac{dV}{dt} & = I_{\textrm{app}} - {g_{\textrm{L}}} (V-E_{\textrm{L}}) - {g_{\textrm {K}}}n(V-E_{\textrm{K}}) \\ &\quad {}- {g}_{\textrm{Ca}}m_{\infty}(V) (V-E_{\textrm{Ca}})-{g}_{ \textrm{KCa}}z(V-E_{\textrm{K}}), \end{aligned}$$

51 $$\begin{aligned} \frac{dn}{dt} & = {\phi} \bigl(n_{\infty}(V)-n \bigr)/\tau_{n}(V), \end{aligned}$$

52 $$\begin{aligned} \frac{dCa}{dt} & = \varepsilon ( -\mu I_{\textrm{Ca}}-Ca ), \end{aligned}$$

53 $$\begin{aligned} z&=\frac{Ca}{Ca +1}. \end{aligned}$$

**Fig. 15:**
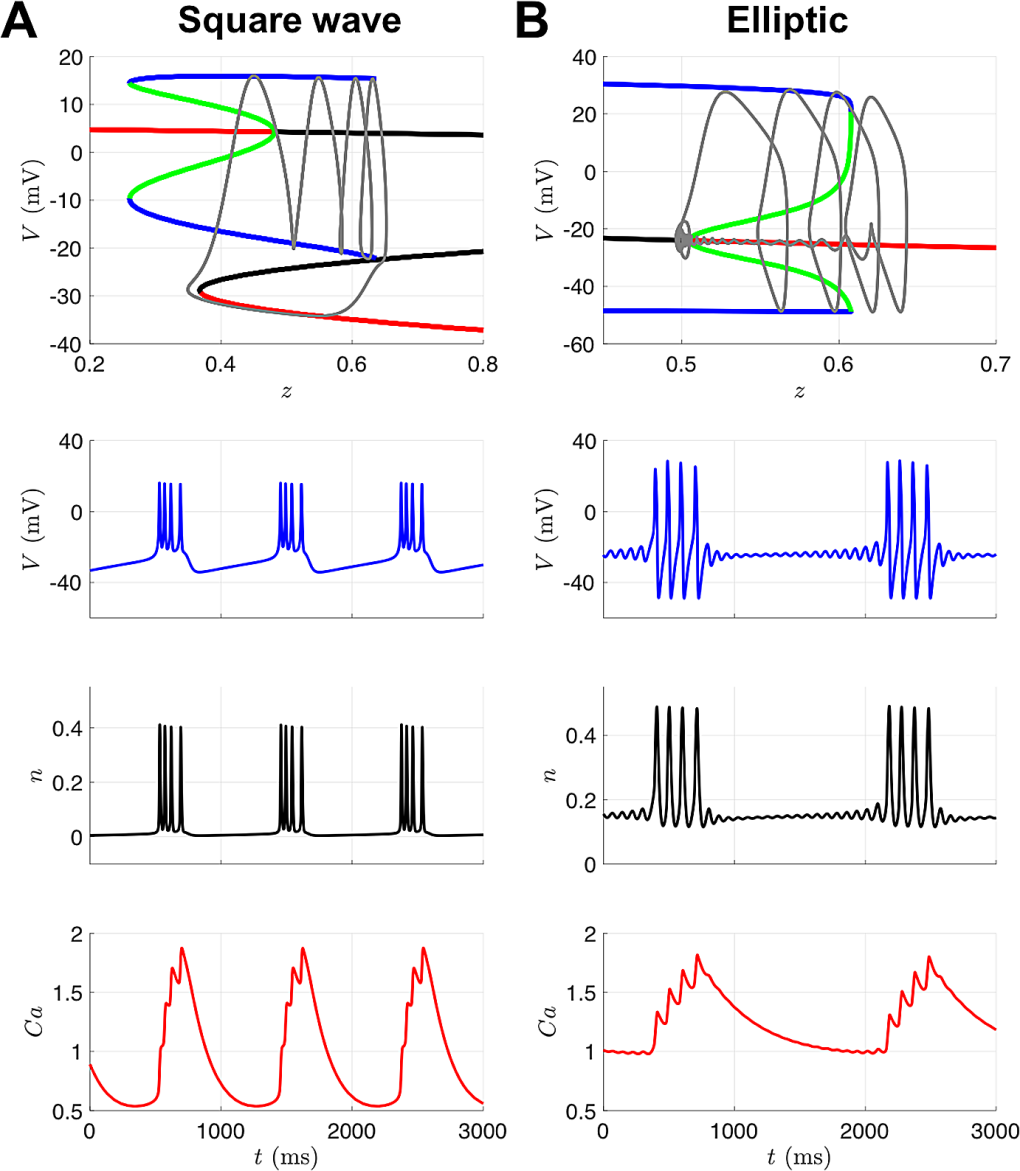
Bursting model bifurcation diagrams and trajectories. The bifurcation diagrams (top row) depict stable fixed points (red), unstable fixed points (black), stable limit cycles (blue), and unstable limit cycles (green) of the fast subsystem $(V,n)$ with bifurcation parameter *z*. The gray curves are the projection of the 3-D burst trajectory (*V*, second row; *n*, third row; *Ca*, fourth row) onto the $(V,z)$ plane, where *z* is a function of *Ca*. **(A)** During the quiescent phase of the burst, *Ca* and therefore *z* are decreasing and the trajectory slowly moves leftward along the lower stable branch of fixed points until reaching the saddle-node bifurcation or “knee”, at which point spiking begins. During spiking, *Ca* and *z* are slowly increasing and the trajectory oscillates while traveling rightward until the stable limit cycle is destroyed at a homoclinic bifurcation and spiking ceases. (**B**) During the quiescent phase of the burst, *z* is decreasing and the trajectory moves leftward along the branch of stable fixed points with small-amplitude decaying oscillations until reaching the Hopf bifurcation, at which point the oscillations grow in amplitude to full spikes. During spiking, *z* is slowly increasing and the trajectory oscillates while traveling rightward until the stable limit cycle is destroyed at a saddle-node of periodic orbits bifurcation and spiking ceases

Bursting can be analyzed mathematically by decomposing models into fast and slow subsystems and applying geometric singular perturbation theory. Several different types of bursters have been classified based on the bifurcation structure of the fast subsystem. In square-wave bursting, the active phase of the burst is initiated at a saddle-node bifurcation and terminates at a homoclinic bifurcation. In elliptic bursting, spiking begins at a Hopf bifurcation and terminates at a saddle-node of periodic orbits bifurcation. The voltage traces produced by these two types of bursting are quite distinct, as shown in Fig. 15.

### Results with UKF

We conducted a set of twin experiments for the bursting model to address the same question as we did for the spiking model: from a voltage trace alone, can DA methods estimate parameters that yield the appropriate qualitative dynamical behavior? Specifically, we simulated data from the square-wave (elliptic) bursting regime, and then initialized the UKF with parameter guesses corresponding to elliptic (square-wave) bursting (these parameter values are shown in Table 5). As a control experiment, we also ran the UKF with initial parameter guesses corresponding to the same bursting regime as the observed data. The observed voltage trace included additive white noise generated following the same protocol as in previous trials. We used 200,001 time points with observations at every 1 ms. Between observations, the system was integrated forward using substeps of 0.025 ms. For the square-wave burster, this included 215 bursts with 4 spikes per burst, and 225 bursts with 2 spikes for the elliptic burster.

**Table 5:** Parameters for bursting in the modified Morris–Lecar model. For square-wave bursting $I_{\textrm{app}}=45$, and for elliptic bursting $I_{\textrm{app}}=120$. All other parameters are the same as in Table 1

	Square-wave	Elliptic
*ϕ*	0.23	0.04
$g_{\textrm{Ca}}$	4	4.4
$V_{3}$	12	2
$V_{4}$	17.4	30
$g_{\textrm{K}}$	8	8
$g_{\textrm{L}}$	2	2
$V_{1}$	−1.2	−1.2
$V_{2}$	18	18
$g_{\textrm{KCa}}$	0.25	0.75
*ε*	0.005	0.005
*μ*	0.02	0.02

**Fig. 16:**
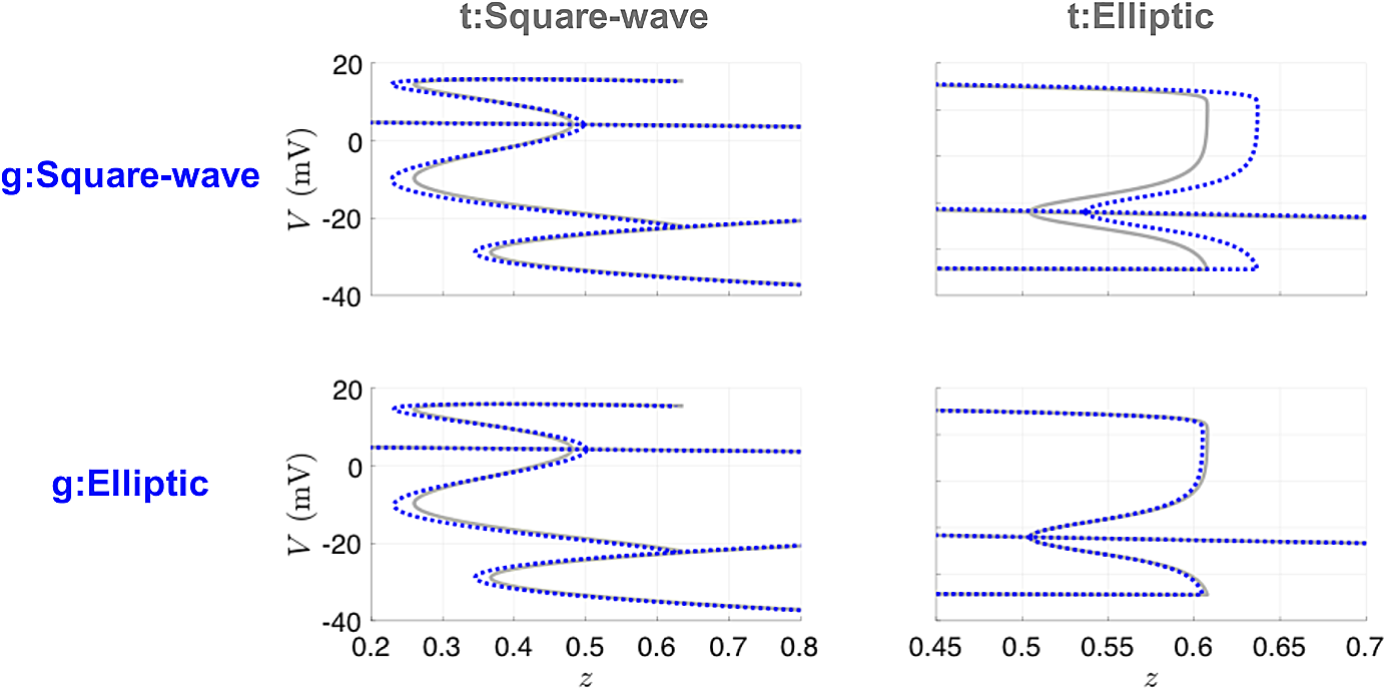
Bifurcation diagrams for UKF twin experiments for the bursting Morris–Lecar model. The gray lines correspond to the true diagrams, and the blue dotted lines correspond to the diagrams produced from the estimated parameters in Table 6

The small parameters *ε* and *μ* in the calcium dynamics equation were assumed to be known and were not estimated by the UKF. Thus, for the bursting model, we are estimating one additional state variable (*Ca*) and one additional parameter ($g_{\textrm{KCa}}$) compared to the case for the spiking model. Table 6 shows the UKF parameter estimates after initialization with either the true parameters or the parameters producing the other type of bursting. The results for either case are quite consistent and fairly close to their true values for both types of bursting. Since small changes in parameter values can affect bursting dynamics, we also computed bifurcation diagrams for these estimated parameters and compared them to their true counterparts. Figure 16 shows that in all four cases, the estimated models have the same qualitative bifurcation structure as the models that produced the data. The recovered parameter estimates were insensitive to the initial conditions for *n* and *Ca*, with 100 different initializations for these state variables sampled from $\mathcal{U}(0,1)$ and $\mathcal {U}(0,5)$, respectively. Note, most predominantly in the top right panel, the location of the bifurcations is relatively sensitive to small deviations in certain parameters, such as $g_{\textrm{KCa}}$. Estimating $g_{\textrm{KCa}}$ is challenging due to the algebraic degeneracy of estimating both terms involved in the conductance $G_{\textrm{KCa}} = g_{\textrm{KCa}}Ca/(Ca+1)$, and the inherent time-scale disparity of the *Ca* dynamics compared to *V* and *n*. If one had observations of calcium, or full knowledge of its dynamical equations, this degeneracy would be immediately alleviated. To address difficulties in the estimation of bursting models, an approach that separates the estimation problem into two stages based on timescales—first estimating the slow dynamics with the fast dynamics blocked and then estimating the fast dynamics with the slow parameters held fixed—has been developed [34].

**Table 6:** UKF parameter estimates for each bursting regime

	t:Square-wave		t:Elliptic	
	g:Square-wave	g:Elliptic	g:Square-wave	g:Elliptic
*ϕ*	0.214	0.215	0.040	0.040
$g_{\textrm{Ca}}$	3.758	3.767	4.396	4.398
$V_{3}$	12.045	12.023	1.603	1.685
$V_{4}$	16.272	16.316	29.582	29.639
$g_{\textrm{K}}$	7.955	7.952	7.866	7.889
$g_{\textrm{L}}$	1.974	1.972	2.015	2.017
$V_{1}$	−1.514	−1.511	−1.120	−1.199
$V_{2}$	17.640	17.624	18.010	18.015
$g_{\textrm{KCa}}$	0.251	0.251	0.767	0.763

### Results with 4D-Var

**Fig. 17:**
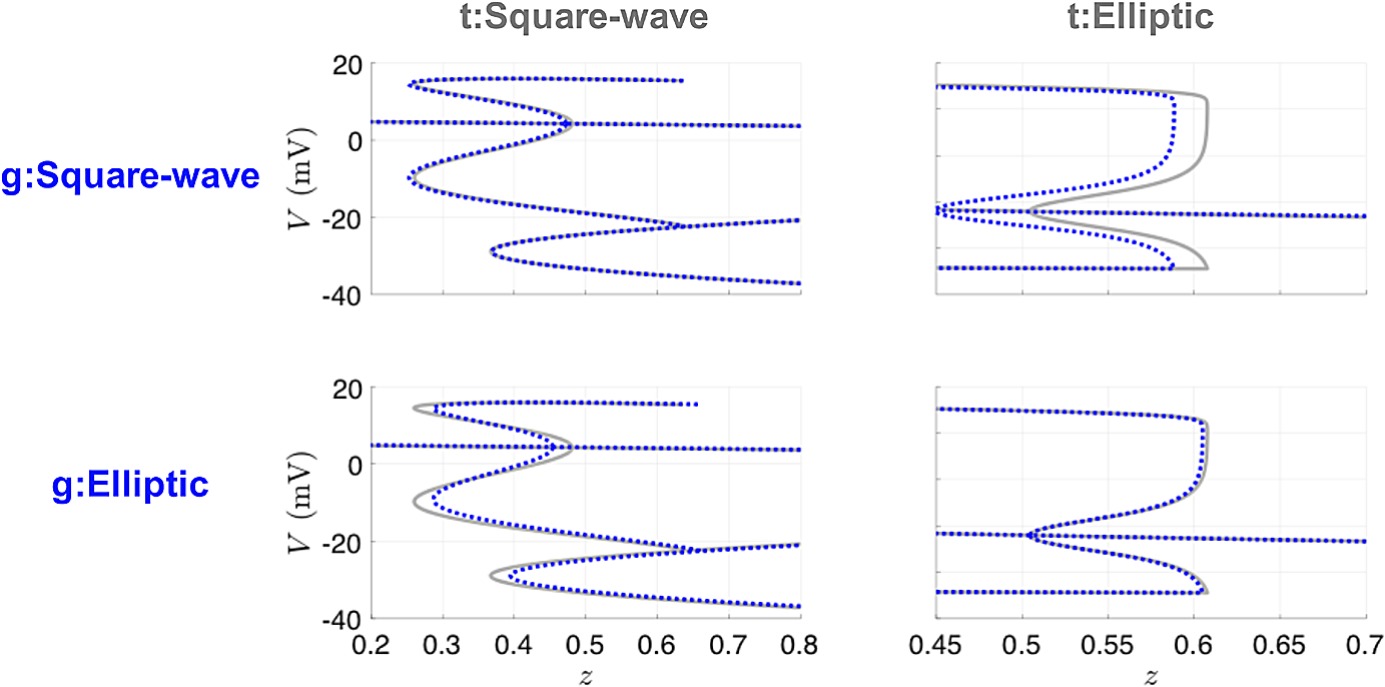
Bifurcation diagrams for 4D-Var twin experiments for the bursting Morris–Lecar model. The gray lines correspond to the true diagrams, and the blue dotted lines correspond to the diagrams produced from the estimated parameters in Table 7

We also investigated the utility of variational techniques to recover the mechanisms of bursting. For these runs, we took our observations to be coarsely sampled at 0.1 ms, and our forward mapping is taken to be one step of modified Euler between observation times, as was the case for our previous 4D-Var Morris–Lecar results. We used 10,000 time points, which is one burst for the square wave burster, and one full burst plus another spike for the elliptic burster. We used the *L-BFGS-B* method [35], as we found it to perform faster for this problem than *fmincon*. This method approximates the Broyden–Fletcher–Goldfarb–Shanno (BFGS) quasi-Newton algorithm using a limited memory (L) inverse Hessian approximation, with an extension to handle bound constraints (B). It is available for Windows through the OPTI toolbox [36] or through a nonspecific operating system MATLAB MEX wrapper [37]. We supplied the gradient of the objective function, but allowed the solver to define the limited-memory Hessian approximation for our 30,012-dimensional problem. The results are captured in Table 7. We performed the same tests with providing the Hessian; however, there was no significant gain in accuracy or speed. The value for $g_{\mathrm{KCa}}$ for initializing with the square wave parameters and estimating the elliptical parameters is quite off, which reflects our earlier assessment for the value in observing calcium dynamics. Figure 17 shows that we are still successful in recovering the true bifurcation structure.

**Table 7:** 4D-Var parameter estimates for each bursting regime

	t:Square-wave		t:Elliptic	
	g:Square-wave	g:Elliptic	g:Square-wave	g:Elliptic
*ϕ*	0.230	0.260	0.037	0.040
$g_{\textrm{Ca}}$	4.009	4.509	4.244	4.412
$V_{3}$	12.009	11.920	6.667	1.971
$V_{4}$	17.437	19.581	32.605	30.026
$g_{\textrm{K}}$	8.006	8.244	9.485	8.002
$g_{\textrm{L}}$	2.003	2.068	1.979	2.009
$V_{1}$	−1.187	−0.627	−1.307	−1.172
$V_{2}$	18.029	18.754	17.469	18.049
$g_{\textrm{KCa}}$	0.250	0.237	0.554	0.741

## Discussion and Conclusions

Data assimilation is a framework by which one can optimally combine measurements and a model of a system. In neuroscience, depending on the neural system of interest, the data we have may unveil only a small subset of the overall activity of the system. For the results presented here, we used simulated data from the Morris–Lecar model with distinct activity based upon different choices for model parameters. We assumed access only to the voltage and the input current, which corresponds to the expected data from a current-clamp recording.

We showed the effectiveness of standard implementations of the Unscented Kalman Filter and weak 4D-Var to recover spiking behavior and, in many circumstances, near-exact parameters of interest. We showed that the estimated models undergo the same bifurcations as the model that produced the observed data, even when the initial parameter guesses do not. Additionally, we are also provided with estimates of the states and uncertainties associated with each state and parameter, but for sake of brevity these values were not always displayed. The methods, while not insensitive to noise, have intrinsic weightings of measurement deviations to account for the noise of the observed signal. Results were shown for mild additive noise. We also extended the Morris–Lecar model to exhibit bursting activity and demonstrated the ability to recover these model parameters using the UKF.

The UKF and 4D-Var approaches implemented here both attempt to optimally link a dynamic model of a system to observed data from that system, with error statistics assumed to be Gaussian. Furthermore, both approaches try to approximate the mean (and for the UKF also the variance) of the underlying, unassumed system distributions. The UKF is especially adept at estimating states over long time courses, and if the algorithmic parameters such as the model error can be tuned, then the parameters can be estimated simultaneously. Therefore, if one has access to a long series of data, then the UKF (or an Unscented Kalman Smoother, which uses more history of the data for each update step) is a great tool to have at one’s disposal. However, sometimes one only has a small amount of time series data, or the tuning of initial covariance, the spread parameter *λ*, and the process noise $Q_{k}$ associated with the augmented state and parameter system becomes too daunting. The 4D-Var approach sets the states at each time point and the parameters as optimization variables, transitioning the estimation process from the one which iterates in time to the one which iterates up to a tolerance in a chosen optimization routine. The only tuning parameters are those associated with the chosen optimization routine, and the weights $Q_{l,l}^{-1} , l\in[1 \dots L]$, for the model uncertainty of the state components at each time. There are natural ways to provide parameter bounds in the 4D-Var framework, whereas this is not the case for the UKF. However, depending upon the implementation choices and the dimension of the problem (which is extremely large for long time series data), the optimization may take a computing time scale of days to yield reasonable estimates. Fortunately, derivative information can be provided to the optimizer to speed up the 4D-Var procedure. Both the UKF and 4D-Var can provide estimates of the system uncertainty in addition to estimates of the system mean. The UKF provides mean and variance estimates at each iteration during the analysis step. In 4D-Var, one seeks mean estimates by minimization of a cost function. It has been shown that for cost functions of the form (36), the system variance can be interpreted as the inverse of the Hessian evaluated at minima of (36), and scales roughly as *Q* for large $Q^{-1}$ [32]. The pros and cons of implementing these two DA approaches are summarized in Table 8.

**Table 8:** Comparison of the sequential (UKF) and variational (4D-Var) approaches to data assimilation

	UKF	4D-Var
Implementation choices	initial covariance ($P_{xx}$)	model uncertainty ($Q^{-1}$)
	sigma points (*λ*)	type of optimizer/optimizer settings
	process covariance matrix (*Q*)	state and parameter bounds
Data requirements	**Pro:** can handle a large amount of data	**Pro:** may find a good solution with a small amount of data
	**Con:** may not find a good solution with a small amount of data	**Con:** cannot handle a large amount of data
Run time	Minutes	Days, hours, or minutes depending on choice of optimizer and settings
Scalability to larger models	Harder to choose Q	Search dimension is (*N* + 1)*L* + *D*
	EnKF may use a smaller number of ensemble members	Sparse Hessian can be exploited during optimization

The UKF and 4D-Var methodologies welcome the addition of any observables of the system, but current-clamp data may be all that is available. With this experimental data in mind, for a more complex system, the number of variables increases, while the total number of observables will remain at unity. Therefore, it may be useful to assess *a priori* which parameters are structurally identifiable and the sensitivity of the model to parameters of interest in order to reduce the estimation state-space [38]. Additionally, one should consider what manner of applied current to use to aid in state and parameter estimation. In the results presented above, we used a constant applied current, but work has been done which suggests the use of complex time-varying currents that stimulate as many of the model’s degrees of freedom as possible [39].

The results we presented are based on MATLAB implementations of the derived equations for the UKF and weak 4D-Var. Sample code is provided in the Supplementary Material. Additional data assimilation examples in MATLAB can be found in [40]. The UKF has been applied to other spiking neuron models such as the FitzHugh–Nagumo model [41]. A sample of this code can be found in [42], as well as further exploration of the UKF in estimating neural systems. The UKF has been used on real data from pyramidal neurons to track the states and externally applied current [43], the connectivity of cultured neuronal networks sampled by a microelectrode array [44], to assimilate seizure data from hippocampal OLM interneurons [15], and to reconstruct mammalian sleep dynamics [17]. A comparative study of the efficacy of the EKF and UKF on conductance-based models has been conducted [45].

The UKF is a particularly good framework for the state dimensions of a single compartment conductance based model as the size of the ensemble is chosen to be $2(L+D)+1$. When considering larger state dimensions, as is the case for PDE models, a more general Ensemble Kalman Filter (EnKF) may be appropriate. An introduction to the EnKF can be found in [46, 47]. An adaptive methodology using past innovations to iteratively estimate the model and measurement covariances *Q* and *R* has been developed for use with ensemble filters [16]. The Local Ensemble Tranform Kalman Filter (LETKF) [48] has been used to estimate the states associated with cardiac electrical wave dynamics [8]. Rather than estimating the mean and covariance through an ensemble, particle filters aim to fully construct the posterior density of the states conditioned on the observations. A particle filter approach has been applied to infer parameters of a stochastic Morris–Lecar model [49], to assimilate spike train data from rat layer V cortical neurons into a biophysical model [50], and to assimilate noisy, model-generated data for other states to motivate the use of imaging techniques when available [51].

An approach to the variational problem which tries to uncover the global minima more systematically has been developed [32]. In this framework, comparing to (36), they define for diagonal entries of $Q^{-1}$ that

$$ Q^{-1}=Q^{-1}_{0}\alpha^{\beta} $$

for $\alpha>1$ and $\beta\geq0$. The model term is initialized as relatively small, and over the course of an annealing procedure, *β* is incremented resulting in a steady increase of the model term’s influence on the cost function. This annealing schedule is conducted in parallel for different initial guesses for the state-space. The development of this variational approach can be found in [52], and it has been used to assimilate neuronal data from HVC neurons [34] as well as to calibrate a neuromorphic very large scale integrated (VLSI) circuit [53]. An alternative to the variational approach is to frame the assimilation problem from a probabilistic sampling perspective and use Markov chain Monte-Carlo methods [54].

A closely associated variational technique, known as “nudging”, augments the vector field with a control term. If we only have observations of the voltage, this manifests as follows:

$$ \frac{dV}{dt} = f^{\star}_{V}(V,\mathbf{q};\boldsymbol {\theta})+ u(V_{\textrm{obs}}-V). $$

The vector field with the observational coupling term is now passed into the strong 4D-Var constraints. The control parameter *u* may remain fixed, or be estimated along with the states [55, 56]. More details on nudging can be found [57]. A similar control framework has been applied to data from neurons of the stomatogastric ganglion [58].

Many other approaches outside the framework of data assimilation have been developed for parameter estimation of neuronal models, see [59] for a review. A problem often encountered when fitting models to a voltage trace is that phase shifts, or small differences in spike timing, between model output and the data can result in large root mean square error. This is less of an issue for data assimilation methods, especially sequential algorithms like UKF. Other approaches to avoid harshly penalizing spike timing errors in the cost function are to consider spikes in the data and model-generated spikes that occur within a narrow time window of each other as coincident [60], or to minimize error with respect to the $dV/dt$ versus *V* phase–plane trajectory rather than $V(t)$ itself [59]. Another way to avoid spike mismatch errors is to force the model with the voltage data and perform linear regression to estimate the linear parameters (maximal conductances), and then perhaps couple the problem with another optimization strategy to access the nonlinearly-dependent gating parameters [3, 61, 62].

A common optimization strategy is to construct an objective function that encapsulates important features derived from the voltage trace, and then use a genetic algorithm to stochastically search for optimal solutions. These algorithms proceed by forming a population of possible solutions and applying biologically inspired evolution strategies to gradually increase the fitness (defined with respect to the objective function) of the population across generations. Multi-objective optimization schemes will generate a “Pareto front” of optimal solutions that are considered equally good. A multi-objective non-dominated sorting genetic algorithm (NSGA-II) has recently been used to estimate parameters of the pacemaker PD neurons of the crab pyloric network [63, 64].

In this paper, we compared the bifurcation structure of models estimated by DA algorithms to the bifurcation structure of the model that generated the data. We found that the estimated models exhibited the correct bifurcations even when the algorithms were initiated in a region of parameter space corresponding to a different bifurcation regime. This type of twin experiment is a useful addition to the field that specifically emphasizes the difficulty of nonlinear estimation and provides a qualitative measure of estimation success or failure. Prior literature on parameter estimation that has made use of geometric structure includes work on bursting respiratory neurons [65] and “inverse bifurcation analysis” of gene regulatory networks [66, 67].

Looking forward, data assimilation can complement the growth of new recording technologies for collecting observational data from the brain. The joint collaboration of these automated algorithms with the painstaking work of experimentalists and model developers may help answer many remaining questions about neuronal dynamics.

### Electronic Supplementary Material

Below is the link to the electronic supplementary material.

Supplementary material (ZIP 7 kB)

## Appendix

**Table A1:** Bounds used during 4D-Var estimation for the results shown in Tables A2 and A3

	Lower Bound	Upper Bound
*ϕ*	0	∞
$g_{\textrm{Ca}}$	0	∞
$V_{3}$	−∞	∞
$V_{4}$	0.1	∞
$g_{\textrm{K}}$	0	∞
$g_{\textrm{L}}$	0	∞
$V_{1}$	−∞	∞
$V_{2}$	0.1	∞

**Table A2:** 4D-Var parameter estimates at the end of the optimization for each bifurcation regime. The loose parameter bounds in Table A1 were used for these trials. Hessian information was not provided to the optimizer

	t:HOPF			t:SNIC			t:HOMO		
	g:HOPF	g:SNIC	g:HOMO	g:HOPF	g:SNIC	g:HOMO	g:HOPF	g:SNIC	g:HOMO
*ϕ*	0.040	0.041	0.040	0.066	0.067	0.066	0.406	0.225	0.229
$g_{\textrm{Ca}}$	4.011	3.959	3.989	4.016	4.035	4.040	8.623	3.992	3.983
$V_{3}$	2.210	13.479	6.284	12.497	12.176	12.102	7.453	14.333	12.197
$V_{4}$	29.917	37.854	32.748	17.589	17.342	16.998	27.569	18.593	17.464
$g_{\textrm{K}}$	8.046	10.857	8.989	8.192	8.057	8.021	9.543	9.213	8.092
$g_{\textrm{L}}$	2.026	1.806	1.959	2.009	2.038	2.067	3.029	1.960	1.990
$V_{1}$	−1.222	−1.188	−1.208	−1.171	−1.165	−1.188	2.604	−1.198	−1.212
$V_{2}$	18.030	17.921	17.979	18.087	18.126	18.148	24.260	18.089	17.985

**Table A3:** 4D-Var parameter estimates at the end of the optimization for each bifurcation regime. The loose parameter bounds in Table A1 were used for these trials. Hessian information was provided to the optimizer

	t:HOPF			t:SNIC			t:HOMO		
	g:HOPF	g:SNIC	g:HOMO	g:HOPF	g:SNIC	g:HOMO	g:HOPF	g:SNIC	g:HOMO
*ϕ*	0.039	0.039	0.039	0.066	0.066	0.066	0.571	0.560	0.549
$g_{\textrm{Ca}}$	3.889	3.889	3.889	4.002	4.002	4.002	831.907	911.887	913.350
$V_{3}$	1.971	1.971	1.971	11.825	11.825	11.825	826.608	896.717	822.366
$V_{4}$	29.533	29.533	29.533	17.071	17.071	17.071	1695.018	1816.501	1813.829
$g_{\textrm{K}}$	8.050	8.050	8.050	7.923	7.923	7.923	847.999	932.249	885.392
$g_{\textrm{L}}$	1.928	1.928	1.928	2.027	2.027	2.027	0.024	0.026	0.118
$V_{1}$	−1.301	−1.301	−1.301	−1.232	−1.232	−1.232	53.706	54.172	53.913
$V_{2}$	17.600	17.600	17.600	18.004	18.004	18.004	75.855	76.135	76.111

**Table A4:** 4D-Var parameter estimates at the end of the optimization for each bifurcation regime. The parameter bounds in Table 4 were used for these trials. Hessian information was provided to the optimizer

	t:HOPF			t:SNIC			t:HOMO		
	g:HOPF	g:SNIC	g:HOMO	g:HOPF	g:SNIC	g:HOMO	g:HOPF	g:SNIC	g:HOMO
*ϕ*	0.039	0.039	0.039	0.066	0.067	0.066	0.230	0.230	0.230
$g_{\textrm{Ca}}$	3.889	3.889	3.889	4.002	4.035	4.002	4.014	4.019	4.014
$V_{3}$	1.971	1.971	1.971	11.825	12.176	11.825	12.321	12.320	12.320
$V_{4}$	29.533	29.533	29.533	17.071	17.342	17.071	17.615	17.633	17.616
$g_{\textrm{K}}$	8.050	8.050	8.050	7.923	8.057	7.923	8.157	8.158	8.157
$g_{\textrm{L}}$	1.928	1.928	1.928	2.027	2.038	2.027	1.996	1.997	1.996
$V_{1}$	−1.301	−1.301	−1.301	−1.232	−1.165	−1.232	−1.154	−1.148	−1.153
$V_{2}$	17.600	17.600	17.600	18.004	18.126	18.004	18.050	18.057	18.050

## List of Abbreviations

DA: data assimilation
PDE: partial differential equation
4D-Var: 4D-Variational
EKF: Extended Kalman Filter
UKF: Unscented Kalman Filter
SNIC: saddle-node on invariant circle
EnKF: Ensemble Kalman Filter
LETK: Local Ensemble Transform Kalman Filter
